# 2-{5-[(*Z*,2*Z*)-2-Chloro-3-(4-nitrophenyl)-2-propenylidene]-4-oxo-2-thioxothiazolidin-3-yl}-3-methylbutanoic Acid as a Potential Anti-Breast Cancer Molecule

**DOI:** 10.3390/ijms23084091

**Published:** 2022-04-07

**Authors:** Kamila Buzun, Agnieszka Gornowicz, Roman Lesyk, Anna Kryshchyshyn-Dylevych, Andrzej Gzella, Robert Czarnomysy, Gniewomir Latacz, Agnieszka Olejarz-Maciej, Jadwiga Handzlik, Krzysztof Bielawski, Anna Bielawska

**Affiliations:** 1 Department of Biotechnology, Faculty of Pharmacy, Medical University of Bialystok, 15-089 Bialystok, Poland; kamila.buzun@umb.edu.pl (K.B.); anna.bielawska@umb.edu.pl (A.B.); 2 Department of Biotechnology and Cell Biology, Medical College, University of Information Technology and Management in Rzeszow, Sucharskiego 2, 35-225 Rzeszow, Poland; dr_r_lesyk@org.lviv.net; 3 Department of Pharmaceutical, Organic and Bioorganic Chemistry, Danylo Halytsky Lviv National Medical University, Pekarska 69, 79010 Lviv, Ukraine; kryshchyshyn.a@gmail.com; 4 Department of Organic Chemistry, Poznan University of Medical Sciences, Grunwaldzka 6, 60-780 Poznan, Poland; akgzella@ump.edu.pl; 5 Department of Synthesis and Technology of Drugs, Faculty of Pharmacy, Medical University of Bialystok, 15-089 Bialystok, Poland; robert.czarnomysy@umb.edu.pl (R.C.); kbiel@umb.edu.pl (K.B.); 6 Department of Technology and Biotechnology of Drugs, Faculty of Pharmacy, Jagiellonian University Medical College, Medyczna 9, 30-688 Cracow, Poland; glatacz@cm-uj.krakow.pl (G.L.); agnieszka.olejarz@uj.edu.pl (A.O.-M.); j.handzlik@uj.edu.pl (J.H.)

**Keywords:** 4-thiazolidinones, breast cancer, apoptosis, autophagy, anticancer agents, chemotherapy, topoisomerase inhibitor, etoposide, 3-MA, ADME-Tox parameters

## Abstract

It was established that the synthesis of hybrid molecules containing a thiazolidinone and a (2*Z*)-2-chloro-3-(4-nitrophenyl)prop-2-ene structural fragments is an effective approach for the design of potential anticancer agents. Given the results of the previous SAR-analysis, the aim of the study was to synthesize a novel 4-thiazolidinone derivative Les-3331 and investigate its molecular mechanism of action in MCF-7 and MDA-MB-231 breast cancer cells. The cytotoxic properties and antiproliferative potential of Les-3331 were determined. The effect of the tested compound on apoptosis induction and mitochondrial membrane potential was checked by flow cytometry. ELISA was used to determine caspase-8 and caspase-9, LC3A, LC3B, Beclin-1, and topoisomerase II concentration. Additionally, PAMPA, in silico or in vitro prediction of metabolism, CYP3A4/2D6 inhibition, and an Ames test were performed. Les-3331 possesses high cytotoxic and antiproliferative activity in MCF-7 and MDA-MB-231 breast cancer cells. Its molecular mechanism of action is associated with apoptosis induction, decreased mitochondrial membrane potential, and increased caspase-9 and caspase-8 concentrations. Les-3331 decreased LC3A, LC3B, and Beclin-1 concentration in tested cell lines. Topoisomerase II concentration was also lowered. The most probable metabolic pathways and no DDIs risk of Les-3331 were confirmed in in vitro assays. Our studies confirmed that a novel 4-thiazolidinone derivative represents promising anti-breast cancer activity.

## 1. Introduction

Cancer is the leading cause of death worldwide. According to the World Health Organization’s (WHO) 2019 estimates, cancer is the first or second leading cause of death among people aged 70 in 112 of the 183 studied countries [1]. Based on the latest WHO data, 2.3 million new cases of breast cancer (BC) occurred among women worldwide in 2020 alone and 685,000 deaths were caused by this disease. By the end of 2020, nearly 8 million women diagnosed with BC in the previous five years were alive worldwide. As statistics show, breast cancer is the most common cancer for women in the world [2].

Molecularly, BC is a heterogeneous disease characterized by hormone receptors (progesterone (PR) and estrogen (ER) receptors) activation, human epidermal growth factor receptor 2 (HER2) activation, and/or *BRCA* mutations. We can distinguish three basic subtypes of BC based on its HER2 and hormone receptor status: luminal (ER-positive and PR-positive), HER2-positive (HER2+), and triple-negative breast cancer (TNBC) [3]. Modern, multidisciplinary therapeutic strategies involve both systematic therapies and locoregional approaches (radiotherapy and surgery). Systematic therapies include chemotherapy, anti-HER2 treatment for HER2+ BC, bone-stabilizing agents, endocrine therapy for hormone receptor-positive breast cancer, poly(ADP-ribose) polymerase inhibitors for patients with *BRCA* mutation, and immunotherapy [4]. Systematic chemotherapy used to date involves the use of cytostatic drugs. Their mechanism of action is based on the apoptosis induction and inhibition of mitosis by disrupting the cell cycle. Chemotherapy options in breast cancer treatment strongly depend on the cancer subtype. Currently, for patients with ER+, PR+, and HER− (after or together with endocrine therapy), intravenous treatment with adriamycin–cyclophosphamide, adriamycin–cyclophosphamide–paclitaxel, or docetaxel–cyclophosphamide can be applied. Chemotherapeutic strategies for patients with HER2+ BC are based on intravenous therapy with paclitaxel–trastuzumab, adriamycin–cyclophosphamide–paclitaxel–trastuzumab ± pertuzumab, or docetaxel–carboplatin–trastuzumab ± pertuzumab. Furthermore, therapy of patients with HER2+, ER+, and PR+ breast cancer includes endocrine treatment as well. TNBC patients are treated with intravenous therapy of adriamycin–cyclophosphamide, adriamycin–cyclophosphamide–paclitaxel, or docetaxel–cyclophosphamide [5]. However, available cytostatic drugs have poor selectivity and a low therapeutic index, leading to a number of side effects. Optimal therapy is different for each patient, based on the diagnosed subtype of cancer, stage of disease and individual preferences of the patient.

Advances in knowledge of molecular biology and cancer genetics are contributing to the search for more effective pharmacotherapeutic approaches based on discoveries related to signal transduction in the cell. Therefore, the search for new anticancer drugs with high therapeutic efficacy and low toxicity is particularly important. Since the 1960s, a significant increase in medicinal chemistry and pharmacology of 4-thiazolidinones has been seen [6,7,8]. Numerous scientific papers, reviews and patents covering novel 4-thiazolidinone analogs have appeared over the last 60 years [9,10,11,12,13]. Compounds belonging to the group of 4-thiazolidinones have demonstrated diversified activity, from anticancer, antimicrobial, antibacterial, and anti-inflammatory to antidiabetic properties [14,15,16]. Among patented analogs and drugs based on 4-thiazolidinones, we can distinguish e.g., antidiabetic Pioglitazone [17], diuretic Etozoline [12], anti-inflammatory Darbufelon [18], or aldose reductase inhibitor Epalrestat [19]. Derivatives of 4-thiazolidinone are a well-known class of patented, leading compounds and drugs, among which antitumor “small molecules” are of special interest [20]. Thus, indisputable evidence of the affinity of 4-thiazolidinone-based derivatives for validated anticancer biotargets, such as TNF-α-TNFRc-1, JSP-1, and antiapoptotic complex Bcl-X_L_-BH3, can be found in the literature [14,21,22]. It is important to note that, in this group of heterocyclic compounds, the most interesting for the design of new biologically attractive molecules are 5-ene-4-thiazolidinones [14]. On the other hand, 5-ene-4-thiazolidinones, as possible Michael acceptors, can react with glutathione and other free thiols within a cell; they are treated as frequent hitters or pan-assay interference compounds (PAINS) with low or insufficient selectivity. This may offer a high probability of polar interactions or hydrogen bonds formation, therefore causing a promiscuous behavior in high throughput screening campaigns that is often not confirmed in experimental studies. Moreover, Michael acceptor functionality, as well as the thesis about low selectivity towards biotargets of rhodanines, must be confirmed experimentally and it cannot be based on just the presence of conjugated α,β-unsaturated carbonyl. Additionally, the positive aspects of Michael acceptors must be considered as well as their multitarget properties [13,23].

As a continuation of our systematic research of 4-thiazolidinone derivatives, we have established that the synthesis of hybrid molecules containing a thiazolidinone and a (2*Z*)-2-chloro-3-(4-nitrophenyl)prop-2-ene structural fragments (*Ciminalum*-thiazolidinone hybrid molecules) is an effective approach for the design of potential anticancer agents. Our systematic SAR-analysis allowed us to establish that the presence of the *Ciminalum* moiety in position 5 of core heterocycle is crucial to the antitumor activity of various 4-thiazolidinone derivatives, including 2,4-thiazolidinediones [24], isomeric 2- [25], and 4-aminothiazolones [26], 2-thioxo-4-thiazolidinones [24], etc. The role of the substituents in position 3 (especially carboxylic groups) of the 4-thiazolidinone core on the level of anticancer cytotoxicity level is also important for further in-depth research. Thus, high cytotoxicity of 5-[(*Z*,2*Z*)-2-chloro-3-(4-nitrophenyl)-2-propenylidene]-2-thioxo-4-thiazolidinone-3-carboxylic acids against gastric cancer (AGS), human colon cancer (DLD-1), and breast cancers (MCF-7 and MDA-MB-231) cell lines, and a fairly wide therapeutic range, were established [24].

Given the results of the previous SAR-analysis, as well as the significant effect of the *Ciminalum*-thiazolidinone hybrids on the breast cancer cells, our current research was aimed at the in-depth study of anti-breast activity of novel derivative Les-3331 (2-{5-[(*Z*,2*Z*)-2-chloro-3-(4-nitrophenyl)-2-propenylidene]-4-oxo-2-thioxothiazolidin-3-yl}-3-methylbutanoic acid).

## 2. Results

### 2.1. Chemistry

**Scheme 1:**
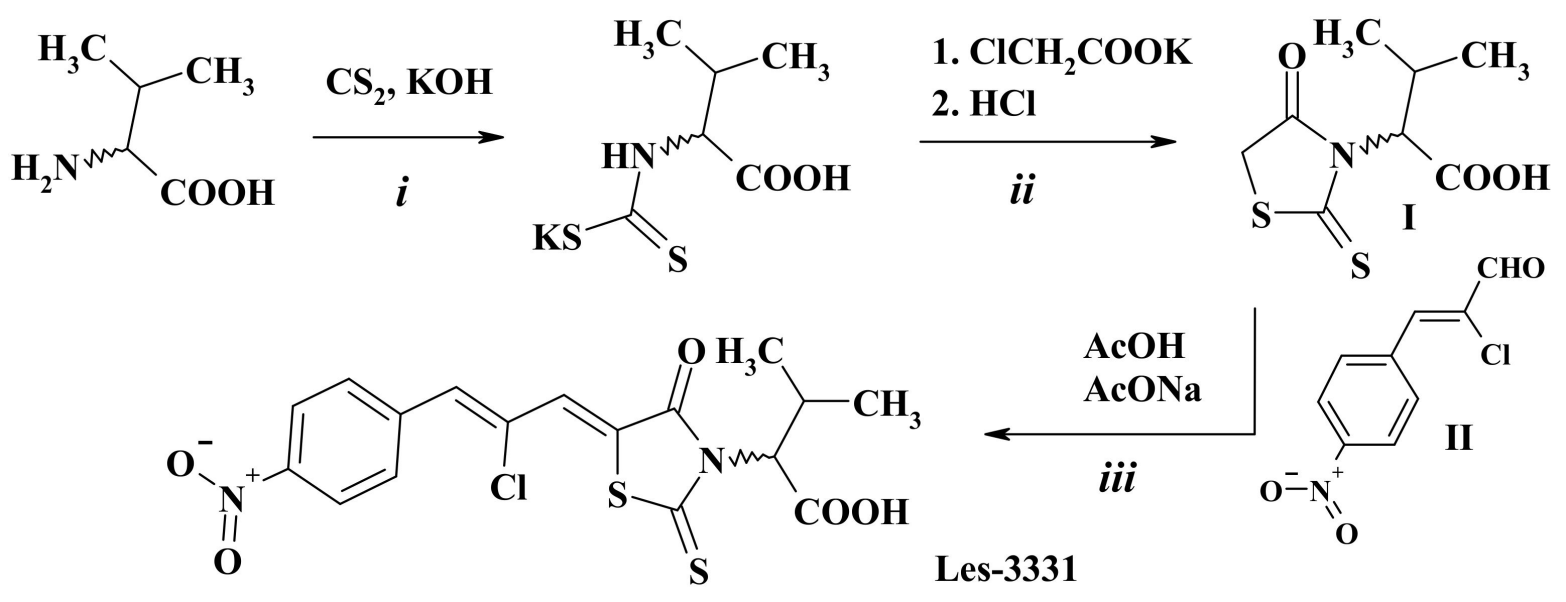
Synthesis of 2-{5-[(*Z*,2*Z*)-2-chloro-3-(4-nitrophenyl)-2-propenylidene]-4-oxo-2-thioxothiazolidin-3-yl}-3-methylbutanoic acid (**Les-3331**). Reagents and conditions: (*i*) *D,L*-Valine (0.03 mol), KOH (0.06 mol), CS_2_ (0.03 mol), H_2_O (30 mL), stirring, RT, 3 h; (*ii*) 1. ClCH_2_COOK (0.03 mol), stirring, RT, 30 min, 2. 2N HCl to pH = 2.0, heating to 90°C, 30 min, 70%; (*iii*) comp. I (0.01 mol), (2Z)-2-chloro-3-(4-nitrophenyl)prop-2-enal **II** (0.010 mol), AcONa (0.01), AcOH (20 mL), reflux, 3 h, 64%.

Target compound Les-3331 was synthesized via the Knoevenagel condensation of 3-methyl-2-(4-oxo-2-thioxothiazolidin-3-yl)-butanoic acid **I** and (2*Z*)-2-chloro-3-(4-nitrophenyl)prop-2-enal **II** in the presence of sodium acetate under reflux in acetic acid (Scheme 1). The starting 3-methyl-2-(4-oxo-2-thioxothiazolidin-3-yl)-butanoic acid **I** was obtained according to the dithiocarbamate method of 2-thioxo-4-thiazolidinones (rhodanines) synthesis using *D,L*-valine as a starting compound [27].

The data characterizing synthesized Les-3331 were presented in the experimental part. Analytical and spectral data (Figures S1–S3, Supplementary Materials) confirmed the structure of the synthesized compound. The ^1^H NMR spectrum of the synthesized Les-3331 is characterized by the signals of the *Ciminalum* residue [24] in the form of two singlets at 7.77 and 8.02 ppm for CH=CCl-CH= group, as well as two doublets of the p-nitrophenyl substituent at 8.04 and 8.32 ppm. The substituent at position 3 of the rhodanine core is characterized by a subspectrum in the form of two doublets for methyl groups at 0.75 and 1.19 ppm, a multiplet and a doublet for CH groups at 2.71 (NCH) and 5.28 ppm (CHCOOH). The carboxylic group forms a broad singlet at 13.28 ppm. In the ^13^C NMR spectra of Les-3331 signals of C=O and C=S groups of the core heterocycle were characteristic and appeared at 166.6 and 194.2 ppm, respectively.

**Figure 1:**
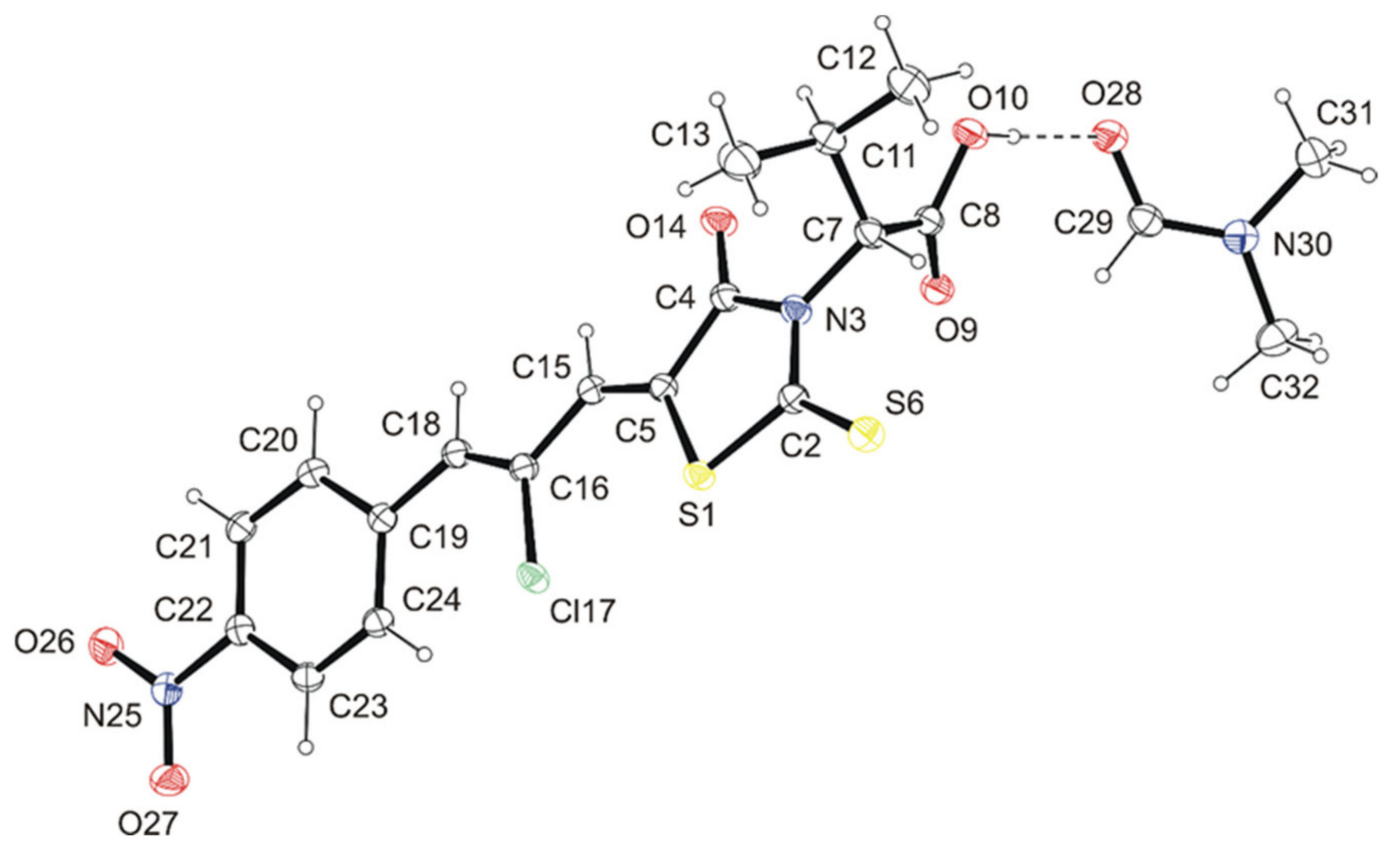
ORTEP view of Les-3331*DMF, showing the atomic labelling scheme. Non-H atoms are drawn as 30% probability displacement ellipsoids and H atoms are drawn as spheres of arbitrary size.

The structure of **Les-3331** was also confirmed by X-ray crystallography (Table S1, Supplementary Materials). The investigated compound has the structure of 2-{5-[(*Z*,2*Z*)-2-chloro-3-(4-nitrophenyl)-2-propenylidene]-4-oxo-2-thioxothiazolidin-3-yl}-3-methylbutanoic acid and crystallizes as dimethylformamide solvate in a molar ratio of 1:1 (Figure 1), which agrees with the previous data for the specified class of compounds [24]. In the salt crystal lattice of Les-3331, solute and solvent molecules related by translation along the *a* axis are linked by hydrogen bonds O10―H10∙∙∙O28, C15—H15∙∙∙O9^i^, C29―H29∙∙∙O9, and C32—H32A∙∙∙O9^iii^ into tapes (Figure S5A, Table S2, Supplementary Materials). The anti-parallel tapes, related by the center of symmetry, then connect by hydrogen bonds C21—H21∙∙∙O26^ii^ into columns (Figure S5B, Table S2, Supplementary Materials).

### 2.2. Biological Studies

**Figure 2:**
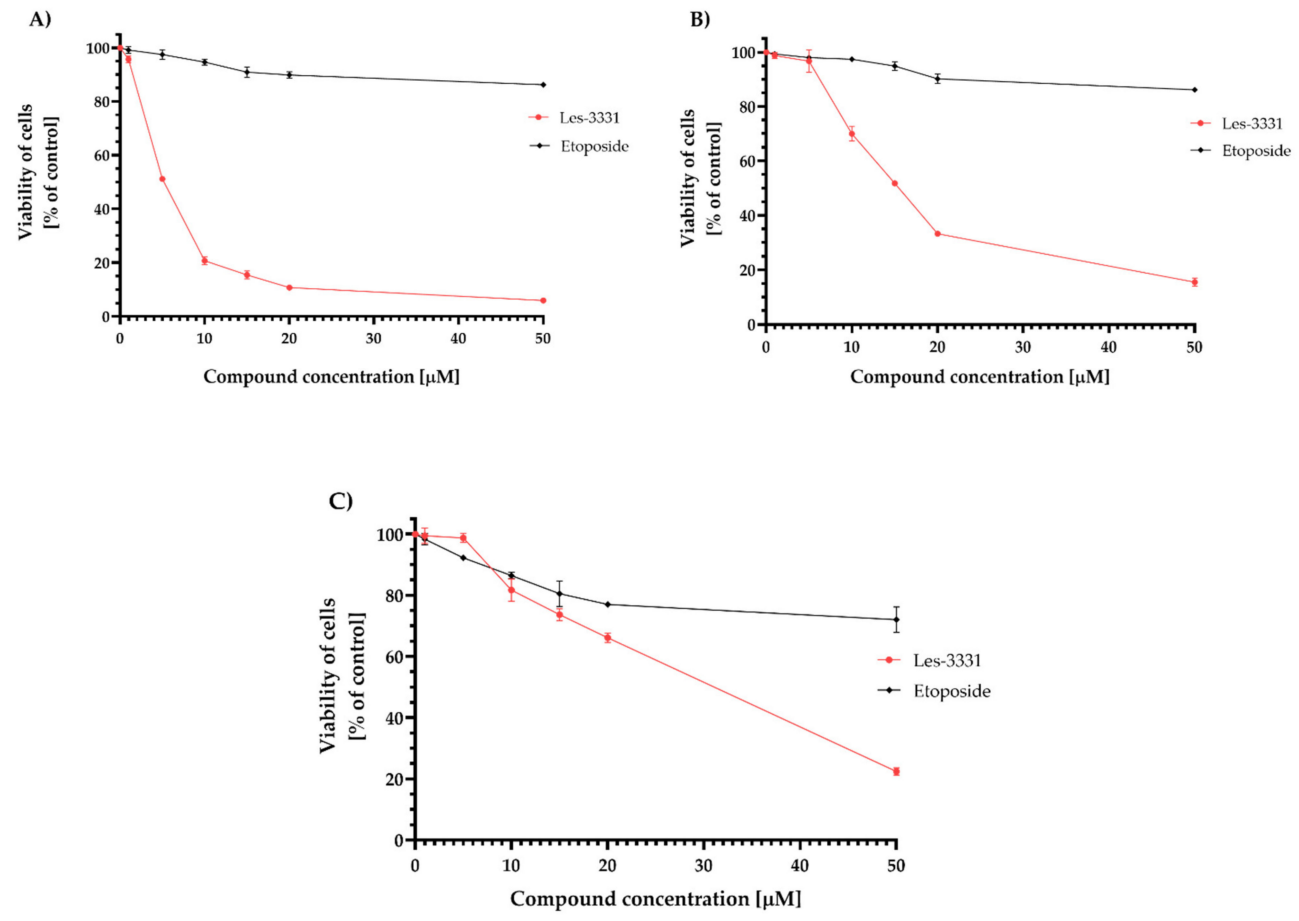
Viability of MCF-7 (**A**), MDA-MB-231 (**B**), and human skin fibroblasts (**C**) incubated for 24 h with Les-3331 and a reference drug (etoposide). Mean ± SD from three independent experiments performed in duplicate is presented.

The influence of Les-3331 on the cell viability of two human breast cancer cell lines and human skin fibroblasts was evaluated using an MTT assay (Figure 2). Cells were incubated with varying concentrations of the tested compound and reference drug (etoposide [28,29,30]) for 24 h.

Based on the results obtained after 24 h incubation with the tested compound and reference drug, we showed that Les-3331 causes a significant reduction in cell viability of human breast cancer cell lines and IC_50_ value for MCF-7 (Figure 2A), and MDA-MB-231 (Figure 2B) cells were 5.02 µM and 15.24 µM, respectively. Etoposide was not as effective in decreasing the viability of human breast cancer cell lines and its IC_50_ values were higher than 50 µM in both cases. Furthermore, as presented in Figure 2C, Les-3331 exhibited lower cytotoxicity against human skin fibroblasts compared to the MCF-7 and MDA-MB-231 cancer cell lines. Its IC_50_ value was 28.52 µM.

**Figure 3:**
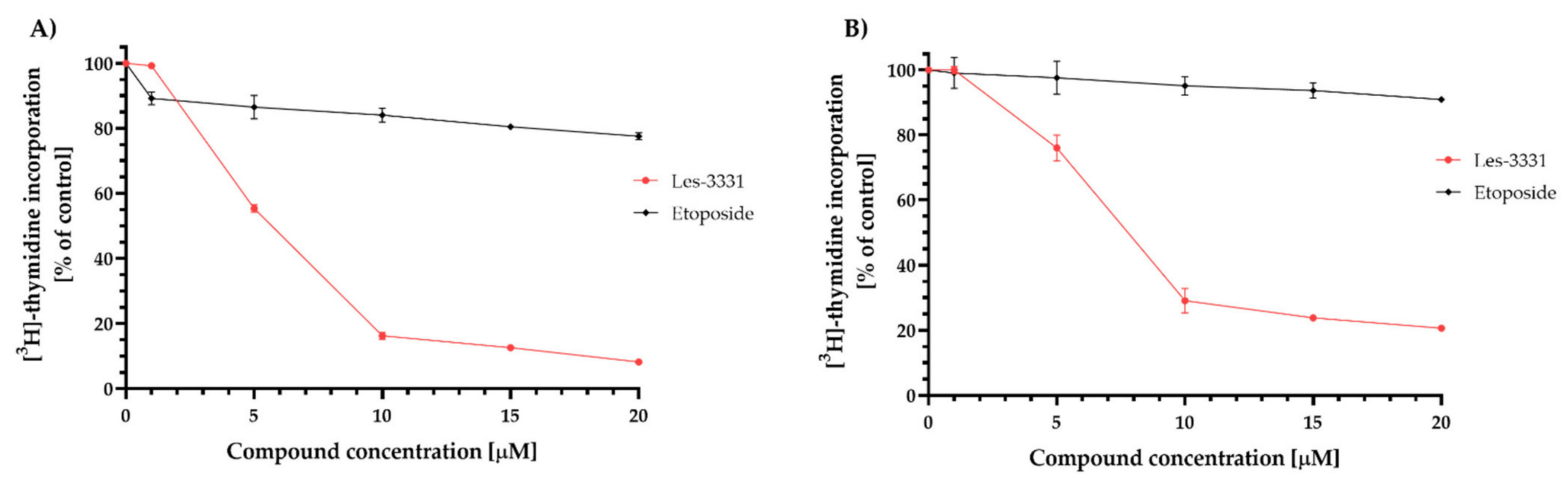
The influence of Les-3331 and reference drug on cell proliferation in MCF-7 (**A**) and MDA-MB-231 (**B**) cancer cell lines. Antiproliferative activity was measured after 24 h incubation of cancer cells with Les-3331 and a reference drug (etoposide) using [^3^H]-thymidine incorporation assay. Mean ± SD from three independent experiments performed in duplicate is presented.

To investigate the effect of novel 2-thioxo-4-thiazolidinone derivative and etoposide on cell proliferation, the level of [^3^H]-thymidine incorporation into DNA of human breast cancer cells was measured. The obtained results are shown in Figure 3.

We demonstrated that exposure of cancer cells to Les-3331 inhibited cell proliferation in a concentration-dependent manner. For MCF-7 cancer cells (Figure 3A), IC_50_ value of the tested compound was 5.54 µM and for MDA-MB-231 cells (Figure 3B), it was 8.01 µM. Etoposide did not exhibit as strong antiproliferative activity as the newly synthesized compound, and its IC_50_ values were higher than 20 µM in both cases.

In order to evaluate the influence of Les-3331 on the apoptosis process in human breast cancer cells, Annexin V binding assay was performed.

**Figure 4:**
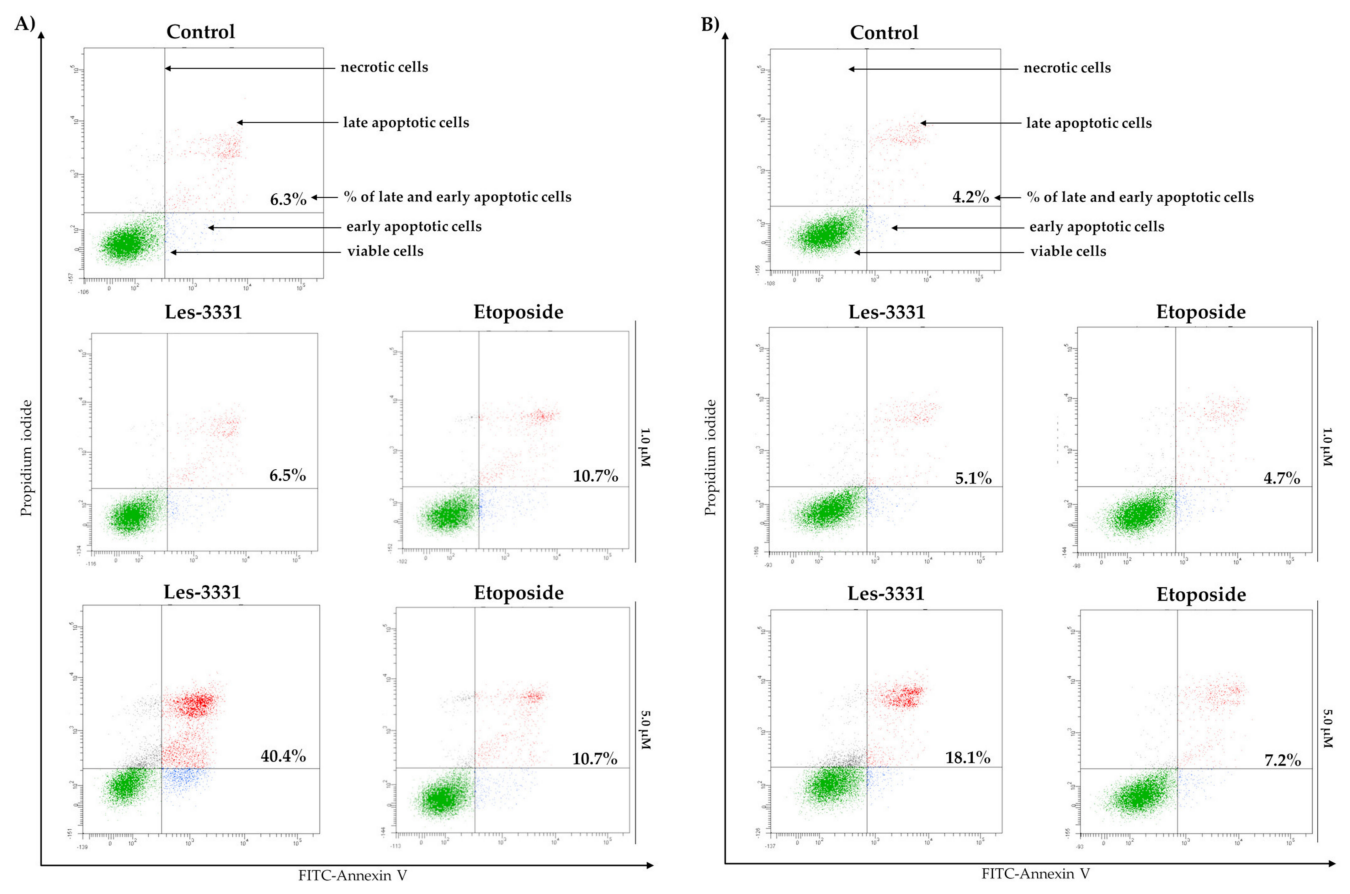
Induction of apoptosis in MCF-7 (**A**) and MDA-MB-231 (**B**) cells incubated with Les-3331 and a reference drug (etoposide) at 1 µM and 5 µM concentration for 24 h. The total number of late and early apoptotic human breast cancer cells is presented as the mean percentage from three independent experiments performed in duplicate.

Analysis of results obtained in Annexin V binding assay revealed that Les-3331 induces apoptosis in a concentration-dependent manner. As shown in Figure 4A, we detected 6.5% of late and early apoptotic MCF-7 cells after a 24 h incubation with 1 µM concentration of Les-3331, whereas after a 24 h incubation of the tested compound with a concentration of five times higher, 40.4% of late and early apoptotic cancer cells were detected. In the case of the MDA-MB-231 cell line (Figure 4B), 5.1% and 18.1% of late- and early-apoptotic cells were detected after 24 h incubation with 1 µM and 5 µM concentration of novel compound, respectively. The reference compound was not as efficient in apoptosis activation as Les-3331. As demonstrated, 10.7% and 4.7% of late and early apoptotic cells were detected after 24 h exposure of MCF-7 (Figure 4A) and MDA-MB-231 (Figure 4B) cells to 1 µM concentration of reference drug, while incubation with 5 µM etoposide revealed 10.7% and 7.2% of apoptotic cells, respectively.

The early stages of apoptosis are correlated with a decrease in mitochondrial membrane potential (ΔΨ_m_) [31]. To investigate the effect of Les-3331 on the intrinsic apoptotic pathway, JC-1 fluorescent dye staining was performed.

**Figure 5:**
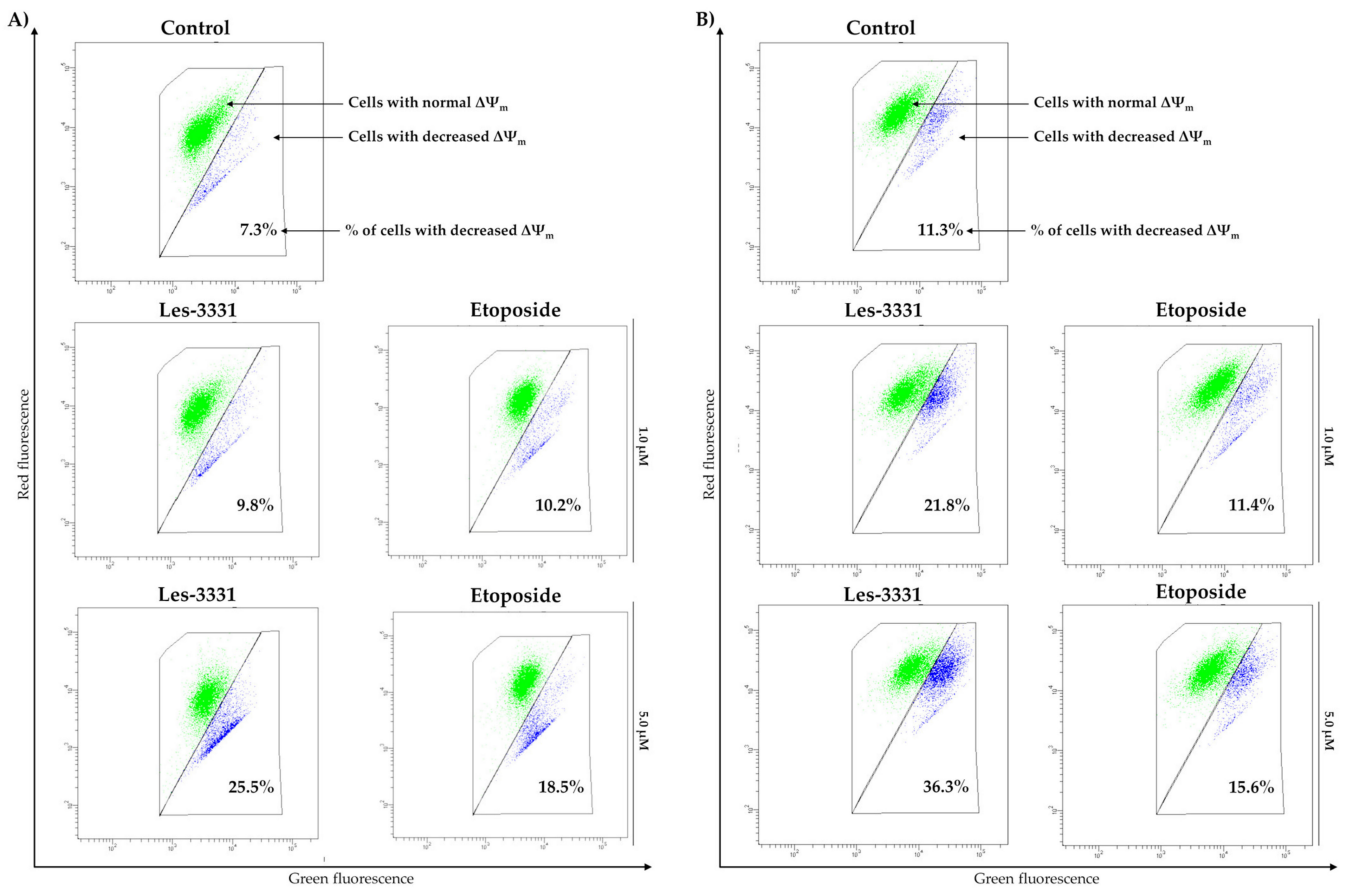
Mitochondrial membrane potential analysis in MCF-7 (**A**) and MDA-MB-231 (**B**) human breast cancer cells incubated with Les-3331 and reference drug (etoposide) at 1 µM and 5 µM concentration for 24 h. The total number of human breast cancer cells with decreased mitochondrial membrane potential is presented as the mean percentage from three independent experiments performed in duplicate.

It was observed that Les-3331 in tested concentrations decreased ΔΨ_m_ in MCF-7 (Figure 5A) and MDA-MB-231 (Figure 5B) human breast cancer cells. After 24 h incubation with 1 µM Les-3331, 9.8% of MCF-7 and 21.8% of MDA-MB-231 cells had decreased ΔΨ_m_, whereas 24 h treatment of cancer cells with a 5 µM concentration of Les-3331 resulted in a decrease in ΔΨ_m_ in 25.5% (MCF-7) and 36.3% (MDA-MB-231) of cells. The weaker effect was observed after incubation with the reference drug (etoposide) in 1 µM and 5 µM concentrations. As demonstrated, 10.2% of MCF-7 (Figure 5A) and 11.4% of MDA-MB-231 (Figure 5B) cells had decreased ΔΨ_m_ after incubation with 1 µM etoposide, while treatment of cancer cells with a reference drug concentration of five times higher resulted in a reduction in ΔΨ_m_ in 18.5% and 15.6% of cells, respectively.

Activation of the intrinsic apoptotic pathway by the newly synthesized compound can be confirmed by examining its influence on caspase-9 (Casp-9) concentration. We investigated whether apoptosis in tested human breast cancer cell lines in the presence of 1 µM and 5 µM of Les-3331 occurs through the intrinsic pathway.

**Figure 6:**
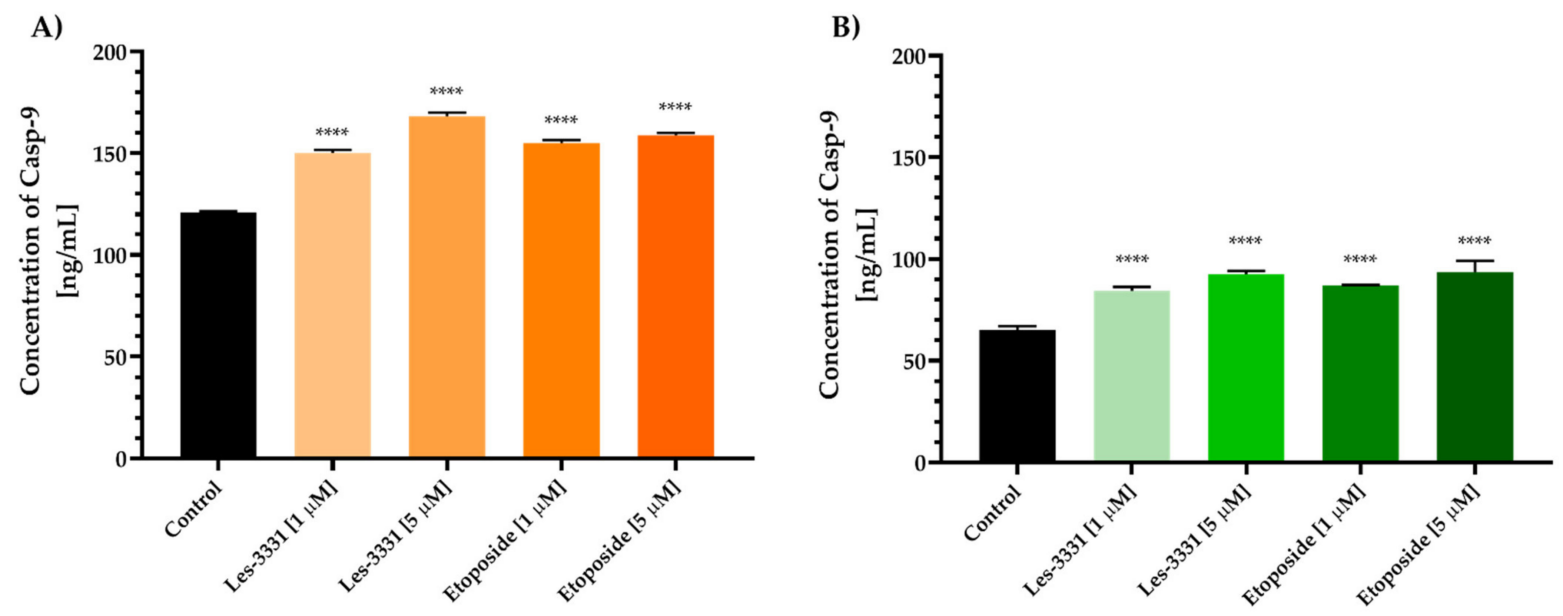
The concentration of caspase-9 in MCF-7 (**A**) and MDA-MB-231 (**B**) human breast cancer cells after 24 h incubation Les-3331 and reference drug (etoposide) at 1 µM and 5 µM concentration. Mean ± SD from three independent experiments performed in duplicate is presented. Statistical significance was calculated using one-way ANOVA with Bonferroni multiple comparison test. Differences were considered statistically significant at **** (*p* ≤ 0.0001).

Based on the obtained results, we showed that Les-3331 causes an increase in Casp-9 concentration. The most significant effect was observed after the exposure of MCF-7 cancer cells to Les-3331 (Figure 6A). After 24 h incubation with 1 µM and 5 µM Les-3331, we detected 150.187 ng/mL and 168.243 ng/mL of Casp-9, respectively, in MCF-7 cell lysates, compared to the control (120.913 ng/mL). The weaker effect was observed after incubation with a reference drug. Compared to the control, the Casp-9 concentration was 154.960 ng/mL (1 µM etoposide) and 158.900 ng/mL (5 µM etoposide). In MDA-MB-231 cells (Figure 6B), the concentration of Casp-9 after treatment with 1 µM and 5 µM Les-3331 was 84.300 ng/mL and 92.523 ng/mL, respectively, compared to the control (65.073 ng/mL). A similar effect was observed after exposure to 1 µM and 5 µM reference drug, where the concentration of Casp-9 was 86.953 ng/mL and 93.620 ng/mL, respectively.

The involvement of Les-3331 in the extrinsic apoptotic pathway can be confirmed through the determination of its influence on caspase-8 (Casp-8) concentration.

**Figure 7:**
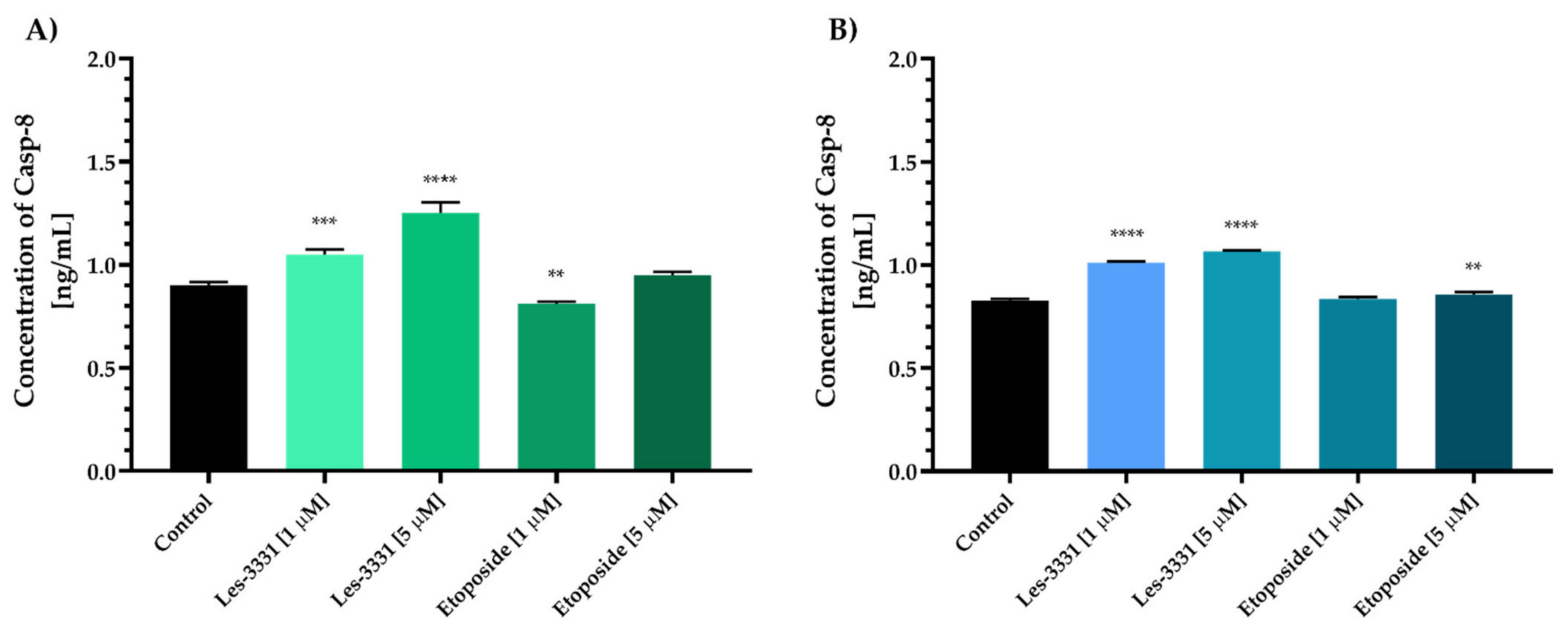
The concentration of Caspase-8 in MCF-7 (**A**) and MDA-MB-231 (**B**) human breast cancer cells after 24 h incubation with Les-3331 and reference drug (etoposide) at 1 µM and 5 µM concentration. Mean ± SD from three independent experiments performed in duplicate is presented. Statistical significance was calculated using one-way ANOVA with Bonferroni multiple comparison test. Differences were considered statistically significant at: ** (*p* ≤ 0.01), *** (*p* ≤ 0.001), **** (*p* ≤ 0.0001).

Studies have shown that Les-3331 caused an increase in Casp-8 concentration. The most significant effect on Casp-8 level was observed after 24 h incubation of MCF-7 cells with Les-3331 (Figure 7A). Compared to the control (0.901 ng/mL), we detected 1.050 ng/mL and 1.253 ng/mL of Casp-8 in cell lysates after exposure of MCF-7 cells to 1 µM and 5 µM Les-3331, respectively. A similar effect of Les-3331 on Casp-8 was shown in MDA-MB-231 cancer cells (Figure 7B). After 24 h incubation of human breast cancer cells with the tested compound, the concentration of Casp-8 was 1.012 ng/mL (1 µM Les-3331) and 1.067 ng/mL (5 µM Les-3331), compared to the untreated control (0.828 ng/mL). The reference compound (etoposide) did not show as strong an effect on Casp-8 concentration as Les-3331. After 24 h exposure to 1 µM etoposide, Casp-8 concentration in cell lysates was 0.812 ng/mL (MCF-7 cells) and 0.836 ng/mL (MDA-MB-231 cells), whereas after treatment with 5 µM etoposide, we detected 0.951 ng/mL and 0.857 ng/mL of Casp-8 in MCF-7 and MDA-MB-231 cells, respectively. 

The microtubule-associated protein 1A/1B light chain 3A (LC3A) and microtubule-associated protein 1A/1B light chain 3B play (LC3B) an important role in the autophagy process due to their interactions with the autophagosomal membrane [32]. To investigate the influence of Les-3331 on the autophagy process, LC3A concentration was checked.

**Figure 8:**
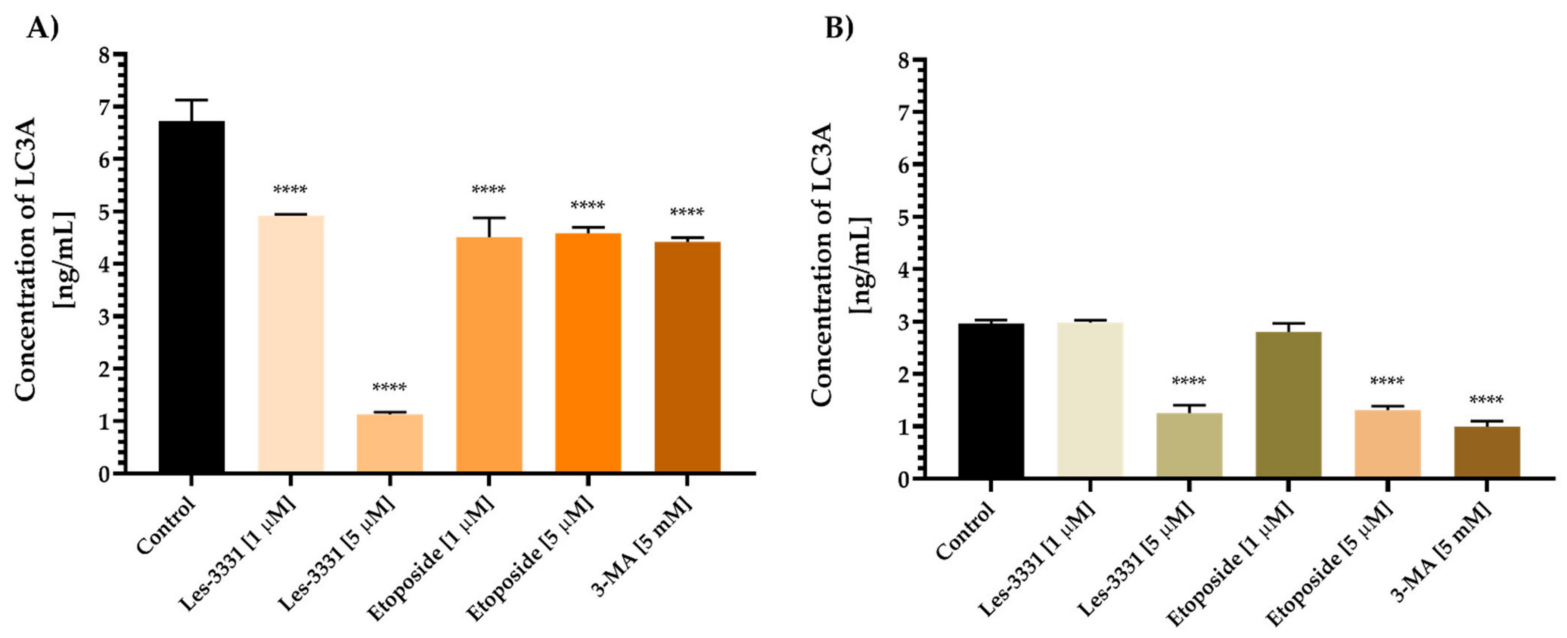
The concentration of LC3A in MCF-7 (**A**) and MDA-MB-231 (**B**) human breast cancer cells after 24 h incubation with Les-3331 and reference drug (etoposide) at 1 µM and 5 µM concentration. As a positive control, 3-Methyladenine (3-MA), a known autophagy inhibitor, in 5 mM concentration was used. Mean ± SD from three independent experiments performed in duplicate is presented. Statistical significance was calculated using one-way ANOVA with Bonferroni multiple comparison test. Differences were considered statistically significant at: **** (*p* ≤ 0.0001).

MCF-7 and MDA-MB-231 human breast cancer cell lines were exposed to Les-3331 in two concentrations, 1 and 5 µM, for 24 h. As demonstrated in Figure 8, a decrease in LC3A concentration was observed after the incubation of cancer cells with a newly synthesized compound and reference drugs. The concentration of LC3A in untreated control MCF-7 cells was 6.726 ng/mL. The exposure of MCF-7 to 1 and 5 µM Les-3331 (Figure 8A) resulted in a decrease in LC3A concentration to 4.917 ng/mL and 1.127 ng/mL, respectively. Furthermore, 24 h incubation with etoposide decreased the LC3A to 4.505 ng/mL (1 µM etoposide) and 4.582 ng/mL (5 µM etoposide). Similarly to MCF-7 cells, incubation of MDA-MB-231 cancer cells with Les-3331 caused a reduction in LC3A concentration (Figure 8B) to 2.982 ng/mL (1 µM Les-3331) and 1.258 ng/mL (5 µM Les-3331), compared to the control (2.965 ng/mL). Etoposide caused a similar effect to Les-3331, decreasing the LC3A concentration to 2.800 ng/mL (1 µM concentration) and 1.313 ng/mL (5 µM concentration). A 24 h incubation of human breast cancer cells with 5 mM 3-Methyladenine (3-MA), a known autophagy inhibitor, resulted in a reduction in LC3A concentration to 4.423 ng/mL (MCF-7 cells) and 0.994 ng/mL (MDA-MB-231 cells). The results obtained after incubation with Les-3331 indicated that in MCF-7 cells, 5 µM Les-3331 showed an almost fourfold stronger inhibitory effect on LC3A concentration (1.127 ng/mL) compared to 5 mM 3-MA (4.423 ng/mL). In MDA-MB-231 cells, 5 µM Les-3331 showed a similar effect on LC3A concentration compared to 5 mM 3-MA: 1.258 ng/mL and 0.994 ng/mL, respectively. 

In order to confirm the above results, the influence of Les-3331 on LC3B concentration was checked.

**Figure 9:**
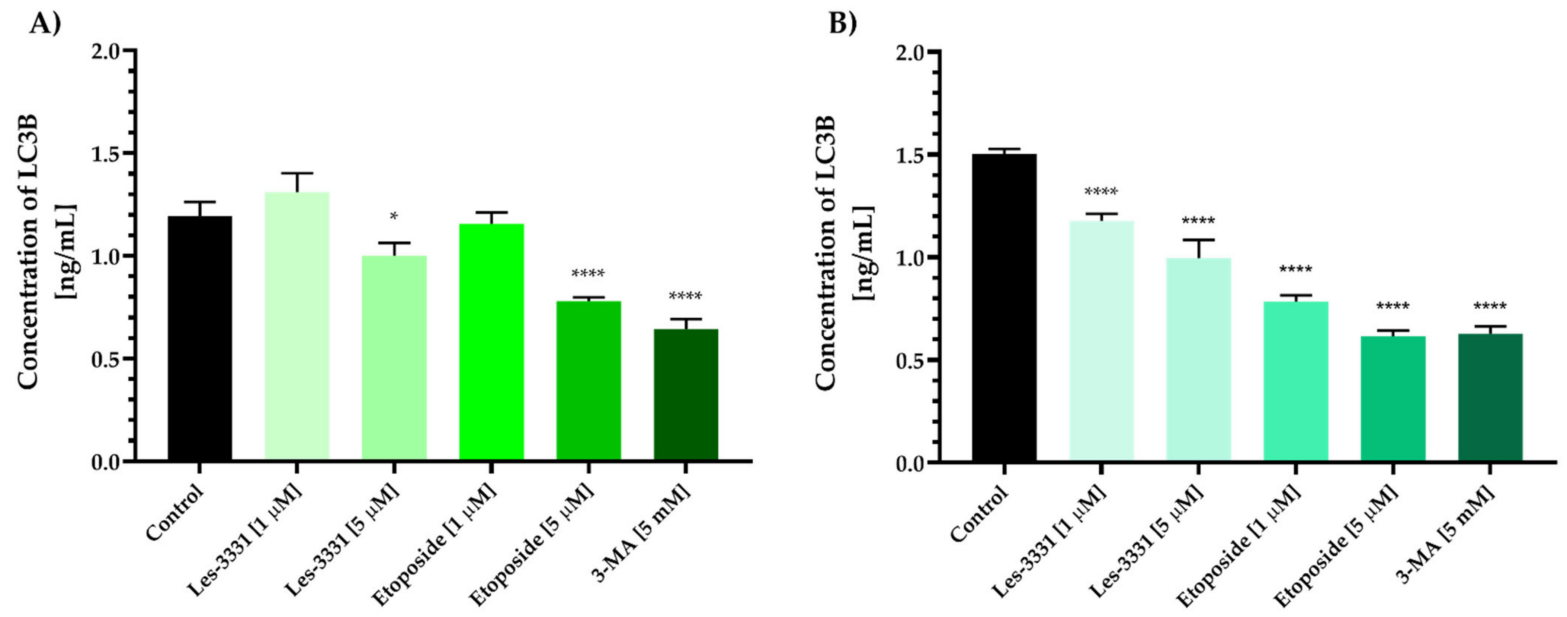
The concentration of LC3B in MCF-7 (**A**) and MDA-MB-231 (**B**) human breast cancer cells after 24 h incubation with Les-3331 and reference drug (etoposide) at 1 µM and 5 µM concentration. As a positive control, 3-Methyladenine (3-MA), a known autophagy inhibitor, in 5 mM concentration was used. Mean ± SD from three independent experiments performed in duplicate is presented. Statistical significance was calculated using one-way ANOVA with Bonferroni multiple comparison test. Differences were considered statistically significant at: * (*p* ≤ 0.05), **** (*p* ≤ 0.0001).

The basal concentration of LC3B in the untreated control group was 1.194 ng/mL in MCF-7 cells (Figure 9A). After 24 h incubation of the tested cells with 5 µM Les-3331, a decrease in LC3B concentration was observed (1.002 ng/mL), while exposure of MCF-7 to 1 µM and 5 µM etoposide reduced LC3B levels to 1.157 ng/mL and 0.779 ng/mL. The strongest effect on LC3B was observed after incubation with 5 mM 3-MA, which reduced the LC3B levels to 0.664 ng/mL, compared to the control and newly synthesized compound. In MDA-MB-231 cells (Figure 9B), Les-3331 decreased the concentration of LC3B to 1.178 ng/mL (1 µM concentration) and 0.996 ng/mL (5 µM concentration), compared to the untreated control cells (1.504 ng/mL). In addition, LC3B levels were also reduced after incubation with 1 µM and 5 µM etoposide, to 0.784 ng/mL and 0.615 ng/mL, respectively. Exposure of MDA-MB-231 cells to 5 mM 3-MA resulted in a decreased LC3B concentration of 0.628 ng/mL. 

Additionally, to confirm that Les-3331 did not induce the autophagy process, its influence on Beclin-1 concentration was analyzed.

**Figure 10:**
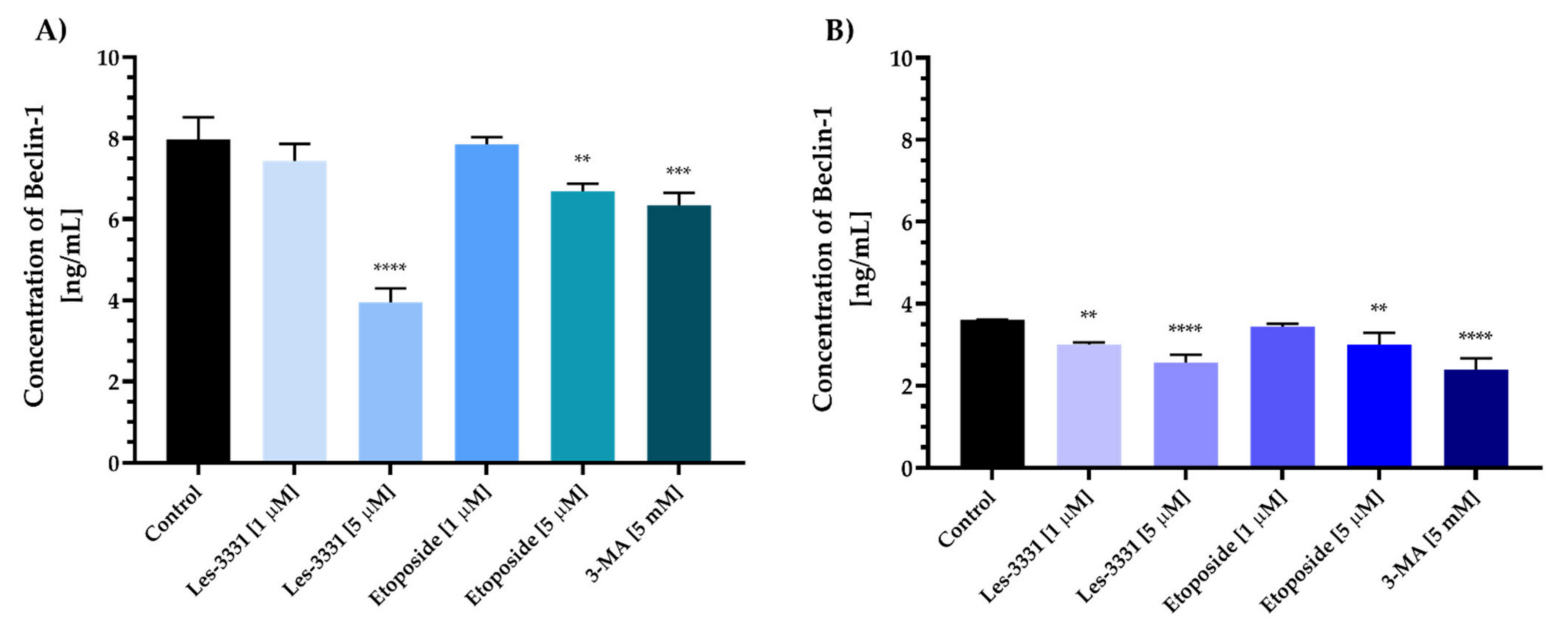
The concentration of Beclin-1 in MCF-7 (**A**) and MDA-MB-231 (**B**) human breast cancer cells after 24 h incubation with Les-3331 and reference drug (etoposide) at 1 µM and 5 µM concentration. As a positive control, 3-Methyladenine (3-MA), a known autophagy inhibitor, in 5 mM concentration was used. Mean ± SD from three independent experiments performed in duplicate is presented. Statistical significance was calculated using one-way ANOVA with Bonferroni multiple comparison test. Differences were considered statistically significant at: ** (*p* ≤ 0.01), *** (*p* ≤ 0.001), **** (*p* ≤ 0.0001).

Based on the obtained results (Figure 10), we showed that 24 h exposure of human breast cancer cell lines to Les-3331 causes a decrease in Beclin-1 concentration in comparison with the control, where 7.965 ng/mL (MCF-7 cells) and 3.605 ng/mL (MDA-MB-231 cells) of Beclin-1 was detected. In MCF-7 cell lysates, a newly synthesized compound reduced the levels of analyzed protein more effectively than etoposide or 3-MA (Figure 10A). We detected 7.435 ng/mL and 3.948 ng/mL of Beclin-1 after incubation with 1 µM and 5 µM Les-3331, while incubation with autophagy inhibitor 3-MA resulted in a reduction in Beclin-1 concentration to 6.344 ng/mL. After exposure to etoposide, Beclin-1 concentration was 7.848 ng/mL (1 µM concentration) and 6.686 ng/mL (5 µM concentration). The weaker effect on Beclin-1 concentration was observed in MDA-MB-231 cells (Figure 10B). Les-3331 slightly reduced Beclin-1 levels and showed similar activity to 3-MA. Amounts of 3.007 ng/mL and 2568 ng/mL of analyzed protein were detected after exposure to 1 µM and 5 µM Les-3331, respectively, compared to 5 mM 3-MA (2.399 ng/mL). The level of Beclin-1 after incubation with 1 µM etoposide is similar to the control group, which was 3.442 ng/mL, whereas the exposure to 5 µM etoposide resulted in a decrease in Beclin-1 to 3.004 ng/mL.

Topoisomerases are responsible for controlling the topology of DNA. The presence and proper functioning of these enzymes are significant for most processes, which occur in the cell. In many cancers, an increase in topoisomerase II activity is observed. The use of chemotherapeutics that inhibit enzyme activity results in DNA strand interruption and consequently leads to cell death [33]. To evaluate the effect of Les-3331 on topoisomerase II (Top II) concentration, an ELISA test was performed.

**Figure 11:**
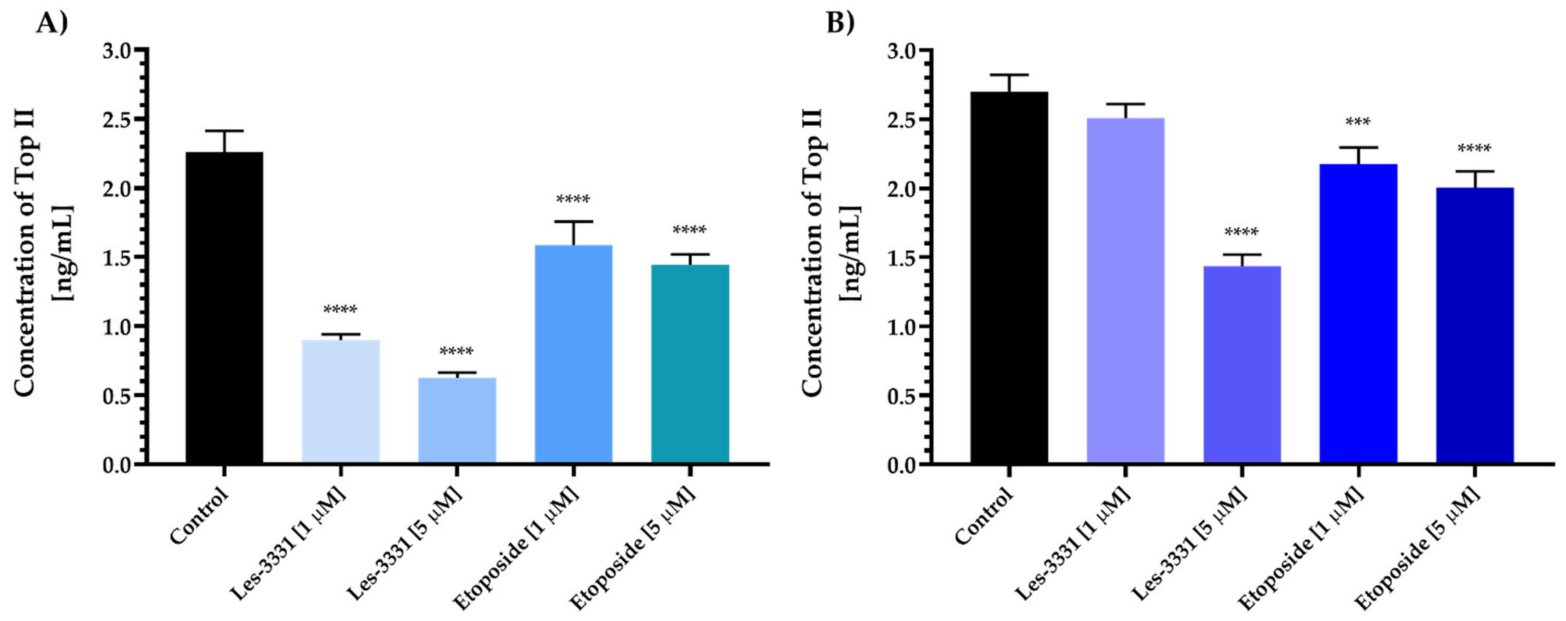
The concentration of topoisomerase II in MCF-7 (**A**) and MDA-MB-231 (**B**) human breast cancer cells after 24 h incubation with Les-3331 and reference drug (etoposide) at 1 µM and 5 µM concentration. Mean ± SD from three independent experiments performed in duplicate is presented. Statistical significance was calculated using one-way ANOVA with Bonferroni multiple comparison test. Differences were considered statistically significant at *** (*p* ≤ 0.001), **** (*p* ≤ 0.0001).

Analysis of the obtained results revealed that Les-3331 causes a reduction in topoisomerase II concentration in both tested human breast cancer cell lines (Figure 11). As shown in Figure 11A, the concentration of Top II was 0.900 ng/mL and 0.625 ng/mL after incubation of MCF-7 cells with 1 µM and 5 µM Les-3331, respectively, compared to the control (2.260 ng/mL of Top II). The weaker effect was demonstrated after exposure to etoposide, where the concentration of Top II was 1.587 ng/mL (1 µM etoposide) and 1.443 ng/mL (5 µM etoposide). In MDA-MB-231 cells (Figure 11B), the concentration of analyzed protein was 2.508 ng/mL after incubation with 1 µM Les-3331. An almost twofold decrease in Top II concentration was observed after treatment with 5 µM Les-3331 (1.436 ng/mL) compared to the control (2.700 ng/mL). Similarly to MCF-7 cells, the weaker effect on Top II concentration was observed after incubation with the reference drug (etoposide) in 1 µM and 5 µM concentrations, where 2.175 ng/mL and 2.003 ng/mL of Top II was detected, respectively.

To confirm the above results, flow cytometric analysis of topoisomerase IIα activity using anti-topoisomerase IIα antibody conjugated with PE was performed.

**Figure 12:**
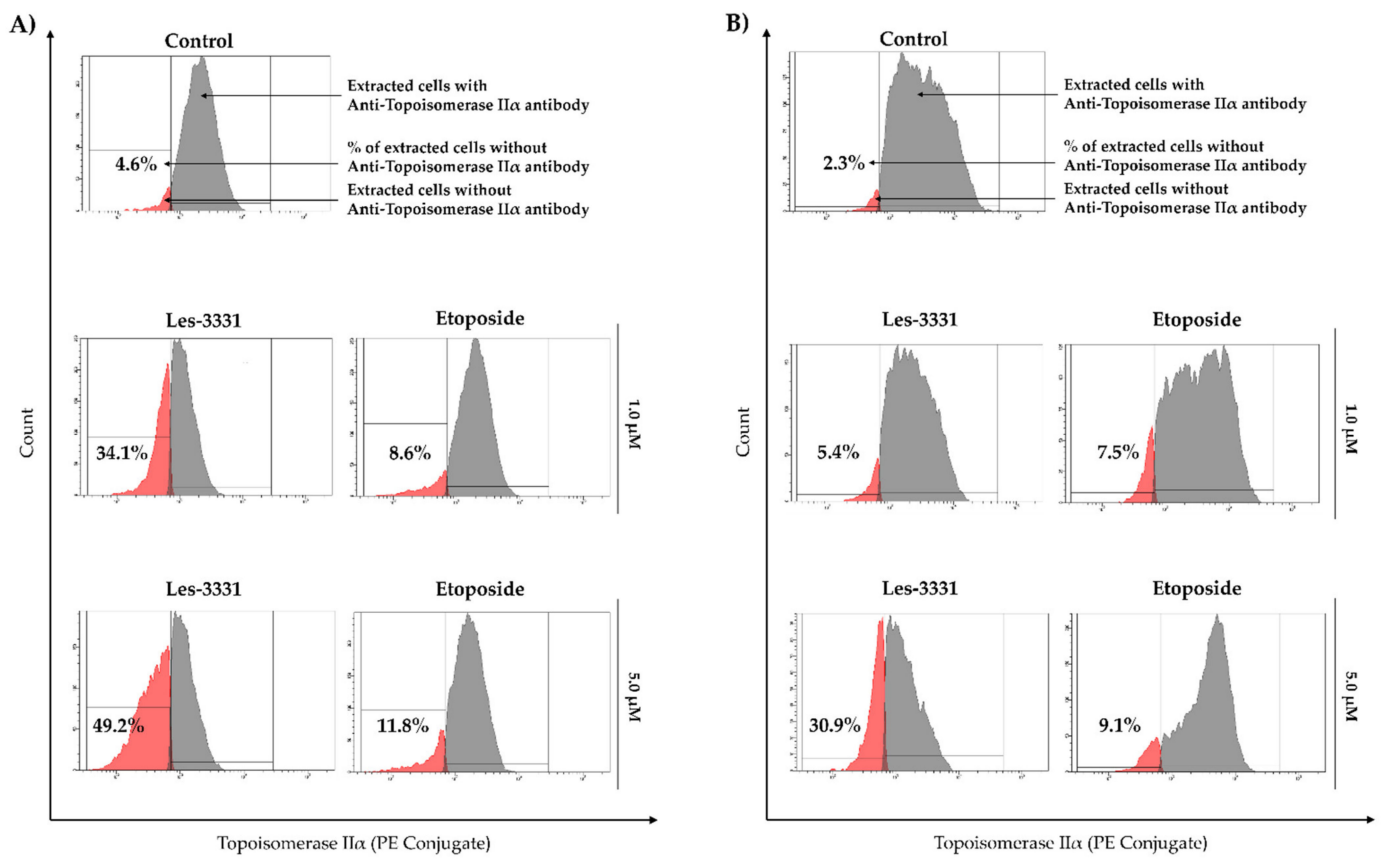
Anti-topoisomerase IIα antibody analysis of MCF-7 (**A**) and MDA-MB-231 (**B**) human breast cancer cells after 24 h incubation with Les-3331 and reference drug (etoposide) at 1 µM and 5 µM concentration. The total number of human breast cancer cells without anti-topoisomerase IIα antibody is presented as the mean percentage from three independent experiments performed in duplicate.

The flow cytometric analysis confirmed the inhibitory effect of Les-3331 on Top II compared to the control and reference drug (Figure 12). It was shown that after a 24 h incubation with Les-3331 in 1 µM concentration, 34.1% of MCF-7 (Figure 12A) and 5.4% of MDA-MB-231 cells (Figure 12B) did not exhibit the presence of anti-topoisomerase IIα antibody. In the 5 µM concentration of the tested compound, that value went up to 49.2% (MCF-7 cells—Figure 12A) and 30.9% (MDA-MB-231 cells—Figure 12B). A weaker effect was observed in 1 µM etoposide, where 8.6% (MCF-7) and 7.5% (MDA-MB-231) cells without anti-topoisomerase IIα antibody were detected. For 5 µM etoposide, 11.8% of MCF-7 and 9.1% MDA-MB-231 without anti-topoisomerase IIα antibody were observed (Figure 12A,B).

### 2.3. Molecular Docking Simulations

**Figure 13:**
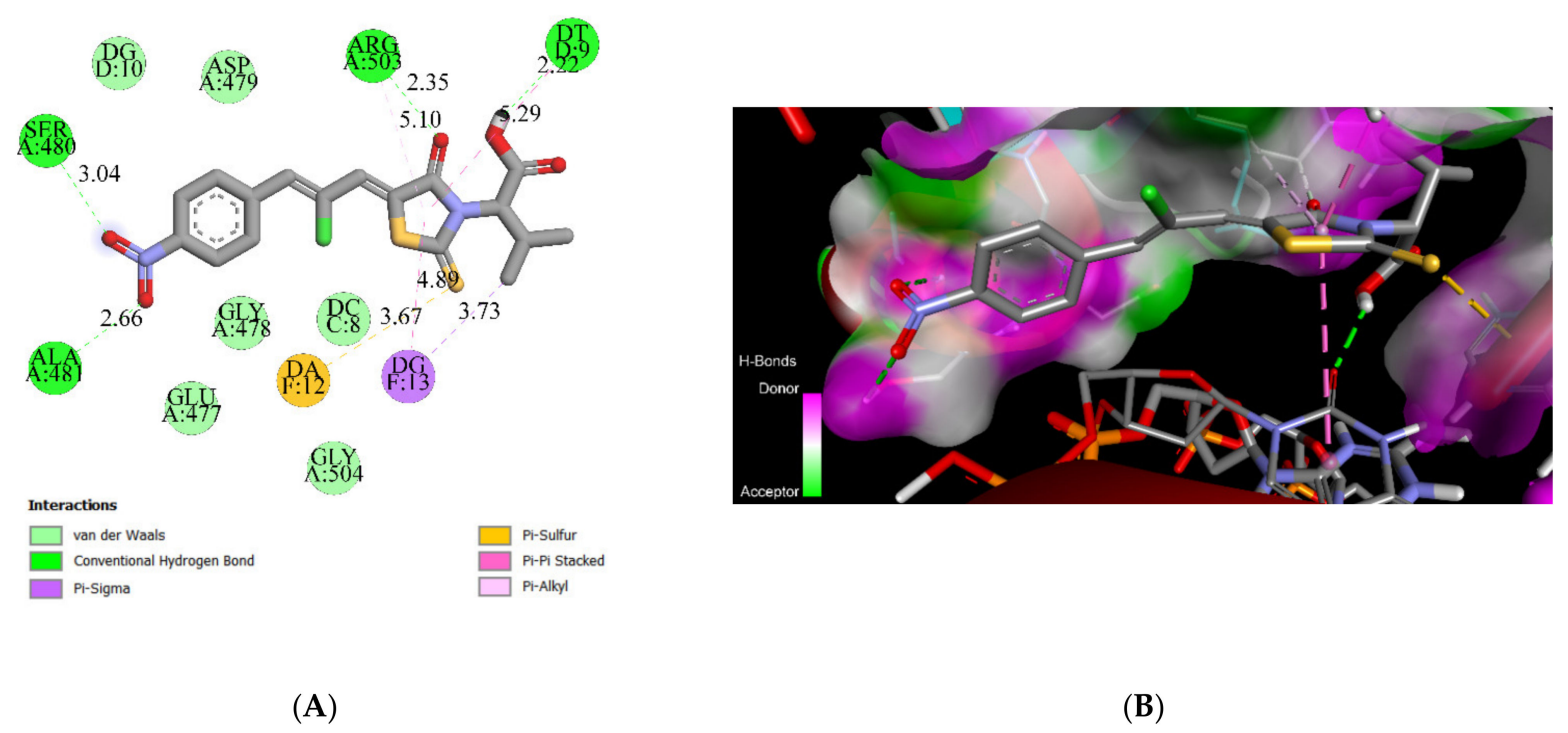
2D (**A**) and 3D (**B**) schemes of the Les-3331–Topoisomerase II complex.

Docking simulations allow for the suggestion that Les-3331 possesses good affinity to Topoisomerase II (Table 1, Figure 13), with the nevertheless summary-binding energy being smaller compared to reference ligand etoposide. The “head” of the molecule incorporates between nucleosides DT9 and DG13 by forming a Pi–Pi-stacked lipophilic interaction. Moreover, the rhodanine core stabilized its position by the hydrogen bond with the Arg503 (2.35 Å). Valine residue of the Les-3331 also connects to DT9 and DG13 by the hydrogen bond (2.22 Å) and Pi–sigma bond (3.73 Å), respectively. Van der Waal forces and Pi–sigma interactions increase the summary energy of the Les-3331–topoisomerase II complex. The nitro group at the tail of the molecule makes two hydrogen bonds with the Ser480 (3.04 Å) and Ala481 (2.66 Å). 

Interaction with topoisomerase II possibly contributes to the summary potency of Les-3331 anti-breast cancer activity.

### 2.4. ADME-Tox In Vitro

Les-3331 was tested in the parallel artificial membrane permeability assay (PAMPA) in order to predict its ability to passively penetrate biological membranes. The obtained data confirmed the low passive permeability of Les-3331. The calculated permeability coefficient (*Pe* = 0.96 × 10^−6^ cm/s) was around sixfold lower than estimated for the one used here as highly permeable reference caffeine (*Pe* = 6.58 × 10^−6^ cm/s).

**Figure 14:**
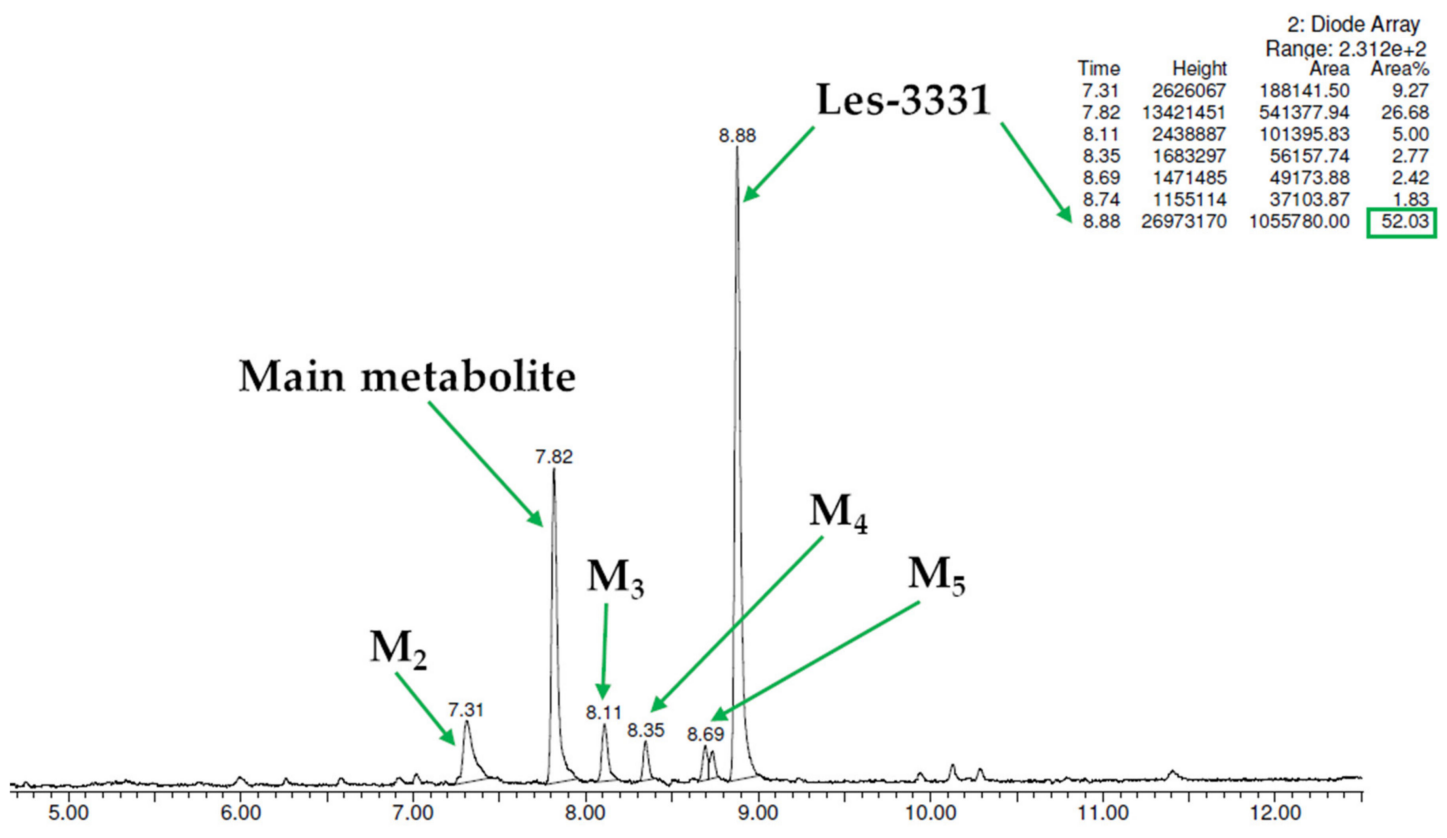
UPLC spectrum of the reaction mixture after 120 min incubation of Les-3331with RLMs.

The metabolic stability of Les-3331 was examined with the use of rat liver microsomes (RLMs). The obtained in vitro data were supported by the prediction of the most probable sites of metabolites performed by MetaSite 8.0.1 software (Figure S6, supporting materials). The in silico analysis showed the sulfur atom of the 2-thioxo-4-thiazolidinone moiety as the most susceptible to the metabolism site of Les-3331. The UPLC analysis after 120 min of incubation with RLMs confirmed that around half of Les-3331 was metabolized into five metabolites M1-M5 (Figure 14). The 52% of compound remained in the reaction mixture and it was a better result than that for the used reference, the unstable drug Verapamil (37.3% remaining). The metabolic stability results were summarized in Table 2.

The most probable main metabolic pathway (M1) was estimated next with the use of MS, MS/MS spectra, and in silico data (see Supplementary Materials, Figures S6–S8). The degradation of the 2-thioxo-4-thiazolidinone moiety was found as the reason for compound decomposition into metabolite M1 and the mass decreasing from *m*/*z* = 427.13 to 397.16. Moreover, the molecular masses of M2-M3 suggest that the M1 metabolite was further metabolized in the reactions of hydroxylation and dehydrogenation (Table 2, Supplementary Materials: Figures S9–S13).

**Figure 15:**
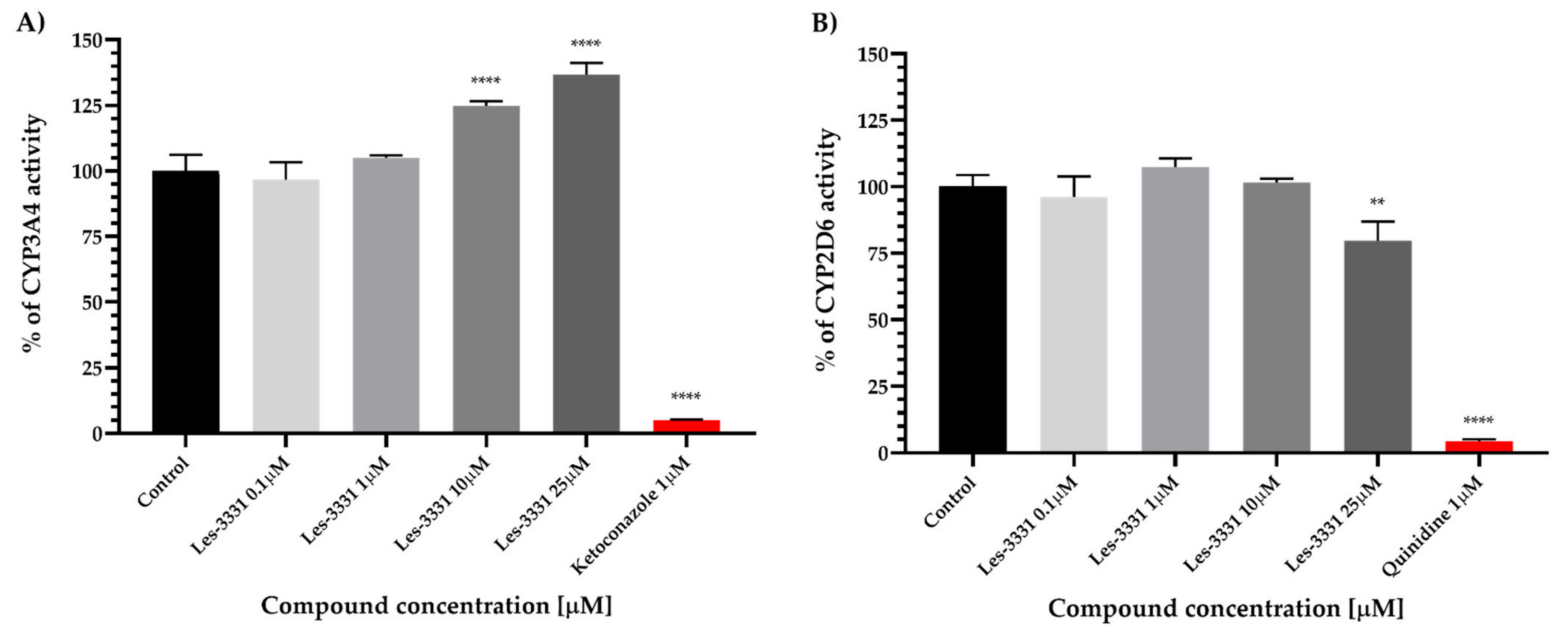
The effect of ketoconazole and Les-3331 on CYP3A4 activity (**A**). The effect of quinidine and Les-3331 on CYP2D6 activity (**B**). Statistical significance was calculated using one-way ANOVA with Bonferroni multiple comparison test. Differences were considered statistically significant at ** (*p* < 0.01), **** (*p* ≤ 0.0001).

To predict or exclude potential drug-drug interactions (DDIs) of Les-3331, we examined its influence on cytochromes CYP3A4 and CYP2D6 activities (Figure 15). These two isoforms were chosen because together they are responsible for the metabolism of more than half of all marketed drugs. The ketoconazole and quinidine (both at 1 µM) were used as the reference inhibitors for CYP3A4 and CYP2D6, respectively. Interestingly, the opposite effects of Les-3331 were shown on the tested CYPs: CYP3A4 was activated at 10 and 25 µM whereas a slight inhibition of CYP2D6 was observed at the highest Les-3331 dose 25 µM. However, when comparing the obtained results to the strong effect of the used references, Les-3331 showed a very low risk of DDIs.

The mutagenicity of Les-3331 was evaluated using Ames MPF protocol with the use of *Salmonella Typhimurium* TA100. This strain is dedicated to the detection of base-pair substitution. The results were compared to the reference mutagen (4-NQO, 26.3 µM). According to the assay protocol, a compound can be considered non-mutagenic if the following criteria are filled: (1) the calculated binomial B-value ≥ 0.99 and (2) the increase in MCB ≥ twofold. Results indicated that Les-3331 was not mutagenic at the concentration of 1 µM based on both criteria. In the case of a higher concentration of 5 µM of Les-3331, the binomial B-value was equal to the breaking point (0.99), but the probable mutagenic effect was not confirmed by the second criterion, i.e., the twofold increase in MCB was not reached by 5 µM of Les-3331 (Figure S14, Supplementary Materials).

## 3. Discussion

Despite the progress in breast cancer therapy, researchers are still looking for novel strategies and compounds, which will be selective and effective against cancer. Chemotherapy represents one of the therapeutic approaches for the treatment of breast cancer. Other approaches include hormone therapy and targeted therapy. 

Apoptosis is a cellular process that plays a pivotal role in carcinogenesis and cancer treatment [35]. Initiator caspases (e.g., caspase-2, -8, -6, -7, -8, -9, and -10) and effector caspases (e.g., caspase-3, -6, and -7) are important players in the initiation and execution of apoptosis. Downregulation, as well as abnormalities in caspase function, may be responsible for decreased programmed cell death. Shen et al. proved that downregulation of caspase-9 in patients with colorectal cancer correlates with poor prognosis [36]. Devarajan et al. demonstrated that downregulation of caspase-3 in breast cancer represents a possible mechanism of chemoresistance [37]. Additionally, cancer cells avoid apoptosis by changing the functions of anti- or pro-apoptotic proteins [38]. Drugs, which are able to induce apoptotic pathways, are worth attention. In this study, we proved that novel Les-3331 induced apoptosis through the extrinsic pathway as well as the intrinsic pathway. It was confirmed by the analysis in Annexin V binding assay, as well as our observation of the increased caspase-8 and caspase-9 concentrations and decreased mitochondrial membrane potential in comparison with the untreated control in both MCF-7 and MDA-MB-231 breast cancer cells.

Autophagy is a multistep lysosomal degradation process, which is classified into macroautophagy, microautophagy, and chaperone-mediated autophagy [32,39]. It possesses dichotomous roles in cancer and acts as a tumor suppressor as well as a mechanism of cell survival. An important role of autophagy in cancer progression was confirmed in breast cancer. Zhao et al. proved that microtubule-associated protein 1A/1B light chain 3B (LC3B) is reconsidered as a marker of autophagy and related to shorter survival in patients with triple-negative breast carcinoma [40]. Autophagy inhibition could be an important strategy for breast cancer treatment. The inhibition of this process may be a useful tool to overcome the drug resistance and increase cancer cell death by apoptosis induction. Many compounds that are able to inhibit autophagy are being tested at different stages of preclinical and clinical trials. Chloroquine, 3-Methyladenine, SAR405, Lys05, ROC-325, Spautin-1, MM124, and MM137 are under investigation in preclinical studies [32,41]. Hydroxychloroquine, verteporfin, and clarithromycin are tested in clinical trials. The novel compound (Les-3331) tested in that study inhibited LC3A, LC3B, and Beclin-1 concentrations in both analyzed breast cancer cells. The inhibitory effect was enhanced after increasing the dose of Les-3331.

DNA topoisomerases represent important molecular targets in anticancer therapy. In this research, we proved that the novel compound had the ability to decrease topoisomerase II concentration and the inhibitory effect was stronger than the reference drug, etoposide, which was approved by the FDA for cancer treatment in 1983. Its mechanism of action is based on topoisomerase II poisoning by binding to the Top II-DNA covalent complexes [33].

The performed PAMPA showed low passive permeability of Les 3331. However, regarding the high biological activity of Les-3331 against the tested cell lines, it may be presumed that either the concentration inside the cell reached by passive mechanism is sufficient to induce the changes leading to cell death or an additional active transport mechanism may be involved in its permeability. Les-3331 also showed moderate metabolic stability with the degradation of the 2-thioxo-4-thiazolidinone moiety as the most probable metabolic pathway and no risk of DDIs. Although Les-3331 was safe at a lower concentration (1 µM) in the AMES test, a risk of mutagenic effects at the higher one (5 µM) was shown. These data may suggest possible interactions of Les-3331 with the DNA of the tested breast cancer cells.

## 4. Materials and Methods

### 4.1. General Information

All reagents and solvents were purchased from commercial suppliers and were used directly without further purification. *Ciminalum* was purchased from State Plant for Chemical Reagents STC of Institute for Single Crystals of the NAS of Ukraine. The elemental analyses (C, H, N) were performed using the Perkin–Elmer 2400 CHN analyzer (Perkin–Elmer, Norwalk, CT, USA). NMR spectra were determined with Varian Unity Plus 400 (400 MHz) and Bruker 170 Avance 500 (500 MHz) spectrometers, in DMSO-*d_6_* using tetramethylsilane (TMS) as an internal standard. Melting points were measured on a Kofler hot stage and are uncorrected. LC-MS was performed using a system with an Agilent 1100 Series HPLC equipped with the diode-array detector and Agilent LC\MSD SL mass-selective detector using chemical ionization at atmospheric pressure (APCI).

### 4.2. Synthesis of 2-{5-[(Z,2Z)-2-Chloro-3-(4-nitrophenyl)-2-propenylidene]-4-oxo-2-thioxothiazolidin-3-yl}-3-methylbutanoic Acid (Les-3331)

A mixture of (2Z)-2-chloro-3-(4-nitrophenyl)prop-2-enal (0.01 mol) and 3-methyl-2-(4-oxo-2-thioxothiazolidin-3-yl)-butanoic acid (0.01 mol) in the medium of acetic acid (20 mL) and the presence of sodium acetate (0.01 mol) was refluxed for 3 h. Obtained solid product was collected after cooling by filtration and recrystallized from the mixture DMF-ethanol (1:2). Yield: 64%, mp 240–242 °C. ^1^H NMR (400 MHz, DMSO-*d_6_*): δ (ppm) 0.75 (d, 3H, *J* = 6.8 Hz, CH_3_), 1.19 (d, 3H, *J* = 6.4 Hz, CH_3_), 2.71 (m, 1H, CH), 5.17 (d, 1H, *J* = 7.6 Hz, CH), 7.77 (s, 1H, CH=), 8.02 (s, 1H, CH=), 8.04 (d, 2H, *J* = 8.8 Hz, arom.), 8.32 (d, 2H, *J* = 8.7 Hz, arom.), 13.28 (br.s, 1H, COOH). ^13^C NMR (100 MHz, DMSO-*d_6_*): δ (ppm) 18.9 (CH_3_), 21.6 (CH_3_), 27.2 (CH), 62.1 (CH), 117.0 (C-Cl), 123.9, 129.6 (=CH), 131.0, 132.2 (=CH), 138.9, 139.7, 147.4, 166.6 (C=O), 168.5 (COOH), 194.2 (C=S). LCMS (ESI): *m*/*z* 426.9/428.9 (100%, [M + H]^+^). Anal. Calc. for C_17_H_15_ClN_2_O_5_S_2_: C 47.83%; H 3.54%; N 6.56%. Found: C 47.90%; H 3.45%; N 6.65%.

### 4.3. Crystal Structure Determination of Les-3331*DMF

Single-crystal X-ray data were collected on Rigaku SuperNova, Single source at offset/far, Atlas [42], using graphite-monochromated CuKα radiation (1.54184 Å). Intensity data were corrected for the Lorentz, polarization, and absorption effects [42]. The structure was solved by the dual-space algorithm (SHELXT) [43,44] and refined against F2 for all data (SHELXL) [44,45]. The position of the H atom bonded to the O atom was obtained from the difference Fourier map and was refined freely. The remaining H atoms were positioned geometrically and were refined using a riding model, with C–H: 0.96 Å (CH_3_), 0.98 Å (C*sp^3^*H), 0.95 Å (C*sp^2^*H), and *U*_iso_(H) = 1.2*U*_eq_(C) or 1.5*U*_eq_(C) for methyl H atoms. The methyl groups were refined as rigid groups, which were allowed to rotate. Software used to prepare materials for publication was WINGX [46] and PLATON [47] programs. The molecular illustrations were drawn using ORTEP for Windows [46].

The deposition number CCDC-2129659 for Les-3331 contains supplementary crystallographic data for this paper. These data can be obtained free of charge via www.ccdc.cam.ac.uk/conts/retrieving.html, (accessed on 16 March 2022 or from the Cambridge Crystallographic Data Centre, 12, Union Road, Cambridge CB2 1EZ, UK; Fax: +44 1223 336033).

### 4.4. Cell Culture

Cell culture MCF-7, MDA-MB-231 human breast cancer cells, and fibroblasts skin cells were purchased from the ATCC—American Type Culture Collection. All cell lines were maintained in DMEM (Corning, Kennebunk, ME, USA). The medium was supplemented with 10% fetal bovine serum—FBS (Eurx, Gdansk, Poland) and 1% antimicrobial substances: penicillin–streptomycin (Corning, Kennebunk, ME, USA). The incubator asserted appropriate growth conditions required for these cell lines: 5% of CO_2_, at 37 °C, with the humidity between 90–95%. Cells were seeded in 100 mm round dishes. An amount of 0.05% trypsin containing 0.02% EDTA (Corning, Kennebunk, ME, USA) and phosphate-buffered saline (PBS) without calcium and magnesium (Corning, Kennebunk, ME, USA) was used to detach cells from a plate once 80–90% cell confluence was achieved. In the next step, cells were reseeded in six-well plates (density—5 × 10^5^ of cells per well) in 1 mL of DMEM after a 24 h incubation used in the presented tests.

### 4.5. Cell Viability Assay

To examine the effect of Les-3331 on cell viability, the MTT assay was performed. Etoposide (Sigma-Aldrich, St Louis, MO, USA) was used as a reference drug. Cells, seeded in six-well plates, were incubated for 24 h with serial dilutions of the tested compound and reference drug in duplicates. In the next step, the liquid was aspirated above the cells and cells were washed three times with PBS. Thereafter, 50 µL of 5 mg/mL of MTT (Sigma-Aldrich, St Louis, MO, USA) was added to 1 mL of PBS. After the required time, the MTT solution was removed and resulting formazan crystals were dissolved in DMSO (Sigma-Aldrich, St Louis, MO, USA). The absorbance was measured using Spectrophotometer UV-VIS Helios Gamma (Unicam/ThermoFisher Scientific Inc., Waltham, MA, USA) at a wavelength of 570 nm. The obtained absorbance in untreated control cells was taken as 100%, while the survival of the cells incubated with tested compounds presented as a percentage of the control value [48].

### 4.6. [^3^H]-Thymidine Incorporation Assay

The antiproliferative properties of the newly synthesized compound were investigated through the [^3^H]-thymidine incorporation assay, as described in the literature [49]. Cells culture was exposed to various concentrations of Les-3331 acid and reference drug for 24 h. Thereafter, cells were washed with PBS and 1 mL of fresh medium was added to each well. Then, 0.5 µCi of radioactive [^3^H]-thymidine was appended, and the incubation continued for four hours. After the following incubation, the liquid was aspirated and the plate was placed on ice. Cells were washed twice with 1 mL of 0.05 M Tris-HCl buffer comprising 0.11 M NaCl, then twice after that with 1 mL of 5% TCA acid (Stanlab, Lublin, Poland). Finally, the cells were dissolved with 1 mL of 0.1 M NaOH with 1% SDS (Sigma-Aldrich, St. Louis, MO, USA) at room temperature (RT). The resulting cell lysates were transferred into scintillation vials containing 2 mL of scintillation fluid. The radioactivity was determined using Scintillation Counter 1900 TR, TRI-CARB (Packard, Perkin Elmer, Inc., San Jose, CA, USA). The intensity of DNA biosynthesis in cells was expressed in dpm of radioactive thymidine incorporated in the DNA. The radioactivity observed in untreated control cells was taken as 100%. Values from the tested compounds were expressed as a percentage of the control value [41].

### 4.7. Flow Cytometry Assessment of Annexin V Binding

The effect of Les-3331 and reference drug on the induction of apoptosis process in human breast cancer cell lines was evaluated using a flow cytometer (BD FACSCanto II, Becton Dickinson Biosciences Systems, San Jose, CA, USA) and Annexin V binding Apoptosis Detection Kit II (BD Biosciences, San Diego, CA, USA). The Les-3331 and reference drug were used in 1 µM and 5 µM concentrations. The test was carried out after 24 h incubation with the tested compounds according to the manufacturer’s protocol and was previously described by our research group [50]. Analysis of the obtained results was performed using FACSDiva software (version 6.1.3, BD Biosciences Systems, San Jose, CA, USA). The equipment was calibrated with BD Cytometer Setup and Tracking Beads (BD Biosciences, San Diego, CA, USA).

### 4.8. Mitochondrial Membrane Potential (ΔΨ_m_) Analysis

The JC-1 MitoScreen kit (BD Biosciences, San Diego, CA, USA) was used for the mitochondrial membrane potential (ΔΨ_m_) analysis. The assay was performed using a flow cytometer (BD FACSCanto II, Becton Dickinson Biosciences Systems, San Jose, CA, USA). The Les-3331 and reference drug were used in 1 µM and 5 µM concentrations. After 24 h incubation with tested compounds, the assay was performed as described in the literature [51]. Analysis of the obtained results was performed using FACSDiva software (version 6.1.3, BD Biosciences Systems, San Jose, CA, USA). The equipment was calibrated with BD Cytometer Setup and Tracking Beads (BD Biosciences, San Diego, CA, USA).

### 4.9. Determination of Caspase-8 and Caspase-9, LC3A, LC3B, Beclin-1, and Topoisomerase II Concentration

High sensitivity assay kits (EIAab Science Co., Ltd., Wuhan, China; Abcam plc., Cambridge, United Kingdom; Cloud-Clone Corp., Katy, TX, USA) were used to determine the concentrations of selected proteins in cell lysates after 24 h incubation with novel compound and reference drug in 1 µM and 5 µM concentrations. In brief, after trypsinization, cells were washed thrice with cold PBS and centrifuged at 1000× *g* for 5 min at 4 °C. Then, cells (1.5 × 10^6^) were suspended in lysis buffer for whole-cell lysates. After the second centrifugation, cellular supernatants were frozen immediately at −70 °C. Untreated cancer cells were taken as a control.

The microtiter plates provided in the kits were pre-coated with an antibody specific to the analyzed antigen. The tests were carried out according to the manufacturer’s protocols.

### 4.10. Molecular Docking Studies

Topoisomerase II (PDB code 3QX3) was chosen as target protein for in silico simulations. During the preparing procedures all ligands, cofactors, and water molecules were removed, polar hydrogen atoms were added and nonpolar hydrogen atoms were merged. Moreover, Kollman charges were added and spread over the residues in the prepared pdbqt files of the target protein. For in silico simulations, we used the obtained 3D structure of the Les-3331 obtained from X-ray structure crystallography. Auto Dock Tool was used for calculation of the binding free energy, which includes all types of interaction (hydrogen bonds, lipophilic interaction, Van Der Waals force, etc.) and estimated Inhibition Constant, Ki. Lamarckian Genetic Algorithm (LGA) parameters were used as a default, which includes 50 runs, 300 populations, 2,500,000 energy evaluations, rate of Gene Mutation 0.02, and rate of Crossover 0.8. [52]. Validations of the selected docking were performed using the ability to render the position of reference ligands from X-ray spectrums (RMSD had to be less than 2). Visualization and interpretation of obtained data were performed by the Discovery Studio Visualizer v.21.1.0.20298^®^.

### 4.11. Flow Cytometric Analysis of Anti-Topoisomerase IIα antibody

To confirm the ELISA results, flow cytometric analysis of Top II activity using an anti-topoisomerase IIα antibody conjugated with phycoerythrin (PE) was performed. The Les-3331 and etoposide were used at 1 µM and 5 µM concentrations. Cancer cells were incubated for 24 h with tested compounds and the assay was carried out in accordance with the manufacturer’s protocol. Briefly, cells were centrifuged and resuspended in 4% formaldehyde. Then, cells were incubated at RT for 15 min and subsequently washed with excess PBS and centrifuged. Thereafter, ice-cold 90% methanol was added to the cells and they were incubated for 1 h in an ice bath. Next, cells were washed with excess PBS and centrifuged again. In the meantime, the primary antibody was diluted (1:100) in PBS. Then, cells were resuspended in the prepared primary antibody (100 µL) and incubated at RT, protected from light, for 1 h. Finally, cells were washed and resuspended in PBS (300 µL). The prepared samples were measured immediately. Analysis of the obtained results was performed using a flow cytometer (BD FACSCanto II, Becton Dickinson Biosciences Systems, San Jose, CA, USA) and FACSDiva software (v6.1.3, BD Biosciences Systems, San Jose, CA, USA). The equipment was calibrated with BD Cytometer Setup and Tracking Beads (BD Biosciences, San Diego, CA, USA).

### 4.12. ADME-Tox In Vitro

All in vitro assays used for the evaluation of Les-3331 ADME-Tox parameters were described previously [34,53]. In brief, for the determination of permeability, Pre-coated PAMPA Plate System Gentest^TM^ was purchased from Corning (Tewksbury, MA, USA). The solutions of Les-3331 and the reference caffeine (200 µM) were prepared in PBS buffer (pH = 7.4). PAMPA plates with compounds added to the donor wells were incubated for 5 h at RT. UPLC-MS Waters ACQUITY—TQD system with the TQ Detector (Waters, Milford, MI, USA) was used next for determination of compounds concentrations in donor and acceptor wells, which were required for calculation of permeability coefficient *Pe* according to formulas provided by the manufacturer. 

The metabolic stability was estimated using rat liver microsomes (RLMs) obtained from Sigma-Aldrich (St. Louis, MO, USA). Les-3331 was incubated in the presence of RLMs and NADPH Regeneration System (Promega, Madison, WI, USA) in Tris-HCl buffer (pH 7.4) for 120 min. Cold ethanol was added next to stop the reaction. The reaction mixture was centrifuged. The aforementioned UPLC-MS device was used to analyze the supernatant. MS/MS ion fragment analyses were performed for Les-3331 and the main metabolite. The in silico prediction of possible Les-3331 metabolic pathways was performed by MetaSite 8.0.1. Software (Molecular Discovery Ltd., Hertfordshire, UK). 

For investigation of potential drug-drug interactions, the luminescent CYP3A4 and CYP2D6 P450-Glo assays obtained from Promega^®^ (Madison, WI, USA) were used. Les-3331 was tested in triplicate at the final concentrations from 0.01 to 25 µM according to the protocols provided by Promega^®^. The reference inhibitors were tested at 1 µM. The luminescent signal was measured using a microplate reader, EnSpire PerkinElmer (Waltham, MA, USA).

The mutagenicity was evaluated using Ames microplate fluctuation protocol (MPF) from Xenometrix (Allschwil, Switzerland). The applied *Salmonella Typhimurium* TA100 strain was enabled to detect base-pair substitution (hisG46 mutation, for which target is GGG). Les-3331 was tested independently in two final concentrations, 1 and 5 µM, in triplicate. The reference mutagen 4-NQO was tested at 26.3 µM. The occurrence of revertants was visualized using pH indicator dye, which was present in the bacterial medium. The color changes from violet to yellow were confirmed using measurements of absorbance with a microplate reader, EnSpire, at 420 nm. According to the protocols and data sheets provided by Xenometrix, the medium control baseline (MCB) was calculated first, which refers to the number of revertants observed in the control (growth medium + 1% DMSO) plus standard deviation. Next, the Binomial B-value was calculated for tested compounds which indicates the probability that spontaneous mutations occurred. The fold increase ≥2 over the MCB and/or Binomial B-value ≥ 0.99 are considered as the mutagen alert.

### 4.13. Statistical Analysis

The obtained results are presented as mean ± standard deviation (SD) from three independent experiments performed in duplicate. The statistical analysis was performed using GraphPad Prism version 6.0 (San Diego, CA, USA). The one-way ANOVA with Bonferroni multiple comparison tests was used to show differences between the control and the cancer cells exposed to varying concentrations of novel compound and reference drug. Statistically significant differences were defined at: * (*p* ≤ 0.05), ** (*p* ≤ 0.01), *** (*p* ≤ 0.001), **** (*p* ≤ 0.0001).

## 5. Conclusions

We have shown that novel Les-3331 is cytotoxic towards both tested breast cancer cell lines and induce the extrinsic and intrinsic apoptotic pathways. Furthermore, Les-3331 caused a decrease in LC3A, LC3B, and Beclin-1 concentration. Les-3331 caused a reduction in Top II concentration in both tested human breast cancer cell lines. Furthermore, the most probable metabolic pathways for Les-3331 were found in the model in vitro. In general, this agent displayed a moderate ADMET profile in vitro, including no DDIs risk, and some probability of mutagenic effects observed at the higher concentration, but not at the lower one (1 µM). All data proved that Les-3331 is a promising compound, representing multitargeted potential in breast cancer therapy. The obtained results constitute the basis for further in vivo investigation.

## Figures and Tables

**Table 1:** Binding energies and inhibition constants of the Les-3331 and Topoisomerase II.

Compounds	Topoisomerase II	
	Binding Energy, kcal/mol	Inhibition Constant, Ki, nM
**Les-3331**	−8.79	360.70
**Etoposide**	−11.97	1.69

**Table 2:** The molecular masses of metabolites and proposed metabolic pathways of Les-3331.

Substrate	Molecular Mass (*m*/*z*)	% of the Remaining Substrate	The Retention Time of Metabolite (min.)	The Molecular Mass of the Metabolite (*m*/*z*)	Proposed Metabolic Pathway
**Les-3331**	427.13	52.03	**Main metabolite M1** 7.82	397.16.	decomposition
			**M2** 7.31	413.17	decomposition and hydroxylation
			**M3** 8.11	393.17	decomposition and dehydrogenation
			**M4** 8.35	397.16	decomposition
			**M5** 8.69	*not estimated*	*not estimated*
Verapamil *	454.60	37.28	**M1** 4.94	441.42	demethylation (norverapamil)
			**M2** 3.99	291.35	defragmentation
			**M3** 4.67	441.42	demethylation

* Results revealed in previous studies using the procedure similar to Les-3331 [34].

## Data Availability

The datasets used and/or analyzed during the current study are available from the corresponding author on reasonable request.

## Supplementary Materials

The following supporting information can be downloaded at: https://www.mdpi.com/article/10.3390/ijms23084091/s1.

## Sample Availability

A sample of the compound is available from the authors.

## Abbreviations

3-MA	3-Methyladenine
ANOVA	analysis of variance
BC	breast cancer
Casp-8	caspase-8
Casp-9	caspase-9
DDIs	drug-drug interactions
ELISA	enzyme-linked immunosorbent assay
ER	estrogen receptor
FBS	fetal bovine serum
LC3A	microtubule-associated protein 1A/1B light chain 3A
LC3B	microtubule-associated protein 1A/1B light chain 3B
MCB	medium control baseline
MPF	microplate fluctuation protocol
MTT	3-(4,5-dimethylthiazol-2-yl)-2,5-diphenyl-2H-tetrazolium bromide
PAMPA	parallel artificial membrane permeability assay
PBS	phosphate-buffered saline
PE	phycoerythrin
PR	progesterone receptor
RLMs	rat liver microsomes
RT	room temperature
TCA	trichloroacetic acid
TMS	tetramethylsilane
TNBC	triple-negative breast cancer
Top II	topoisomerase II
UPLC	ultra-performance liquid chromatography
WHO	World Health Organization
ΔΨ_m_	mitochondrial membrane potential
