# Supplementary material for: 2-{5-[(Z,2Z)-2-Chloro-3-(4-nitrophenyl)-2-propenylidene]-4-oxo-2-thioxothiazolidin-3-yl}-3-methylbutanoic Acid as a Potential Anti-Breast Cancer Molecule
Source: Int J Mol Sci. 2022 Apr 7;23(8):4091. doi: 10.3390/ijms23084091 (PMC9032233; doi:10.3390/ijms23084091)
Supplement: Supplementary file 1 [file ijms-23-04091-s001.zip › ijms-1664958-supplementary.pdf]

Supplementary Material

2-{5-[(*Z*,2*Z*)-2-Chloro-3-(4-nitrophenyl)-2-propenylidene]-4-oxo-2-thioxothiazolidin-3-yl}-3-methylbutanoic Acid as a Potential Anti-Breast Cancer Molecule

Kamila Buzun [1], Agnieszka Gornowicz [1,*], Roman Lesyk [2,3], Anna Kryshchyshyn-Dylevych [3], Andrzej Gzella [4], Robert Czarnomysy [5], Gniewomir Latacz [6], Agnieszka Olejarz-Maciej [6], Jadwiga Handzlik [6], Krzysztof Bielawski [5] and Anna Bielawska [1]

[1] Department of Biotechnology, Faculty of Pharmacy, Medical University of Bialystok, 15-089 Bialystok, Poland; kamila.buzun@umb.edu.pl (K.B.); anna.bielawska@umb.edu.pl (A.B.)
[2] Department of Biotechnology and Cell Biology, Medical College, University of Information Technology and Management in Rzeszow, Sucharskiego 2, 35-225 Rzeszow, Poland; dr_r_lesyk@org.lviv.net
[3] Department of Pharmaceutical, Organic and Bioorganic Chemistry, Danylo Halytsky Lviv National Medical University, Pekarska 69, 79010 Lviv, Ukraine; kryshchyshyn.a@gmail.com
[4] Department of Organic Chemistry, Poznan University of Medical Sciences, Grunwaldzka 6, 60-780 Poznan, Poland; akgzella@ump.edu.pl
[5] Department of Synthesis and Technology of Drugs, Faculty of Pharmacy, Medical University of Bialystok, 15-089 Bialystok, Poland; robert.czarnomysy@umb.edu.pl (R.C.); kbiel@umb.edu.pl (K.B.)
[6] Department of Technology and Biotechnology of Drugs, Faculty of Pharmacy, Jagiellonian University, Medical College, Medyczna 9, PL 30-688 Cracow, Poland; glatacz@cm-uj.krakow.pl (G.L.); agnieszka.olejarz@uj.edu.pl (A.O.-M.); j.handzlik@uj.edu.pl (J.H.)
* Correspondence: agnieszka.gornowicz@umb.edu.pl

Table of contents:

1. Copies of ^{1}H, ^{13}C NMR and LC-MS spectra of Les-3331……2-3
2. Crystal structure determination (X-ray) of Les-3311……4-7
3. *In silico* prediction of the most probable sites of metabolism……7
4. MS spectrum and MS ion fragment analyses of Les-3331……8
5. MS spectrum and MS ion fragment analyses of Les-3331 main metabolite M1……9
6. MS spectra of Les-3331 metabolites M2-M5……9-10
7. *In silico* prediction of the most probable sites of the main metabolite M1 hydroxylation……10
8. The AMES test results……11

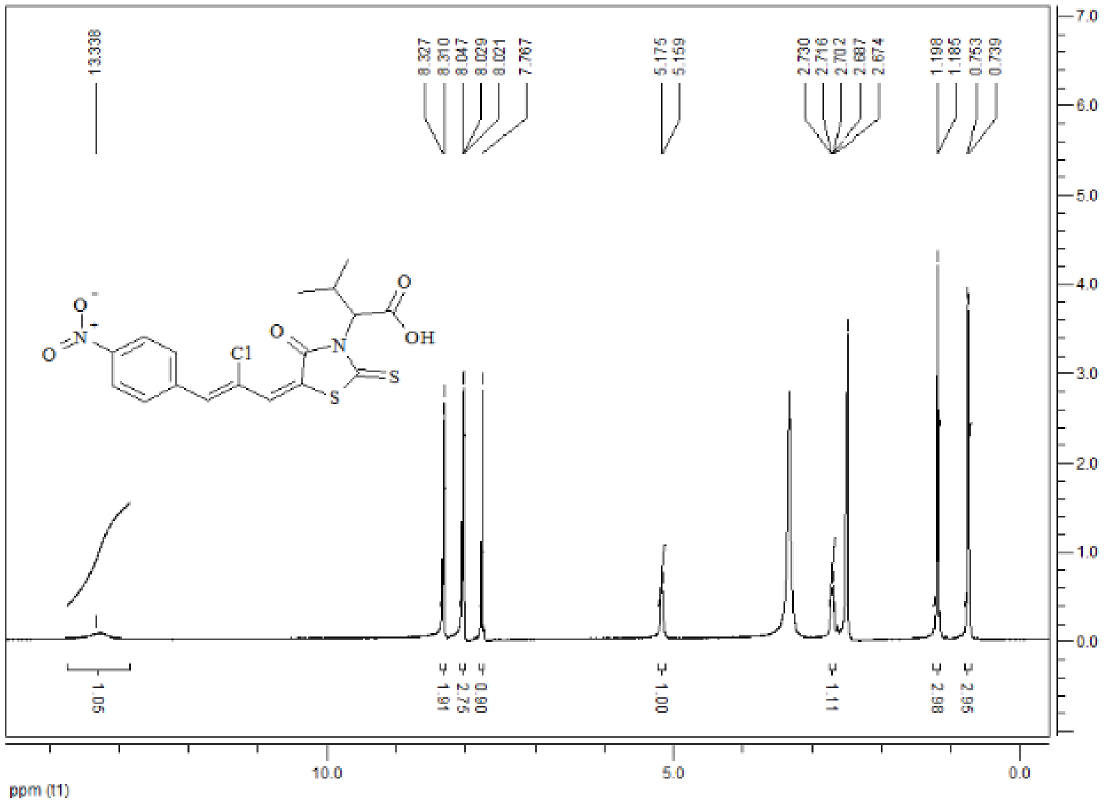

Figure S1. ^{1}H NMR spectrum of Les-3331

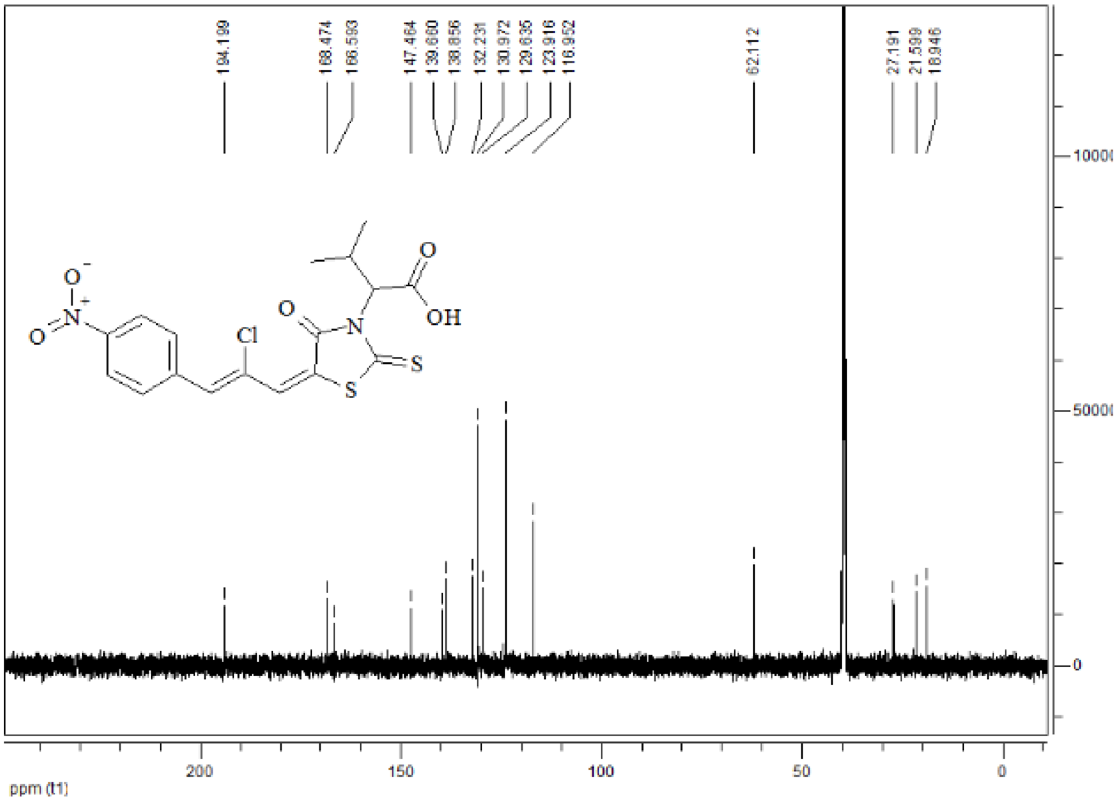

Figure S2. ^{13}C NMR spectrum of Les-3331

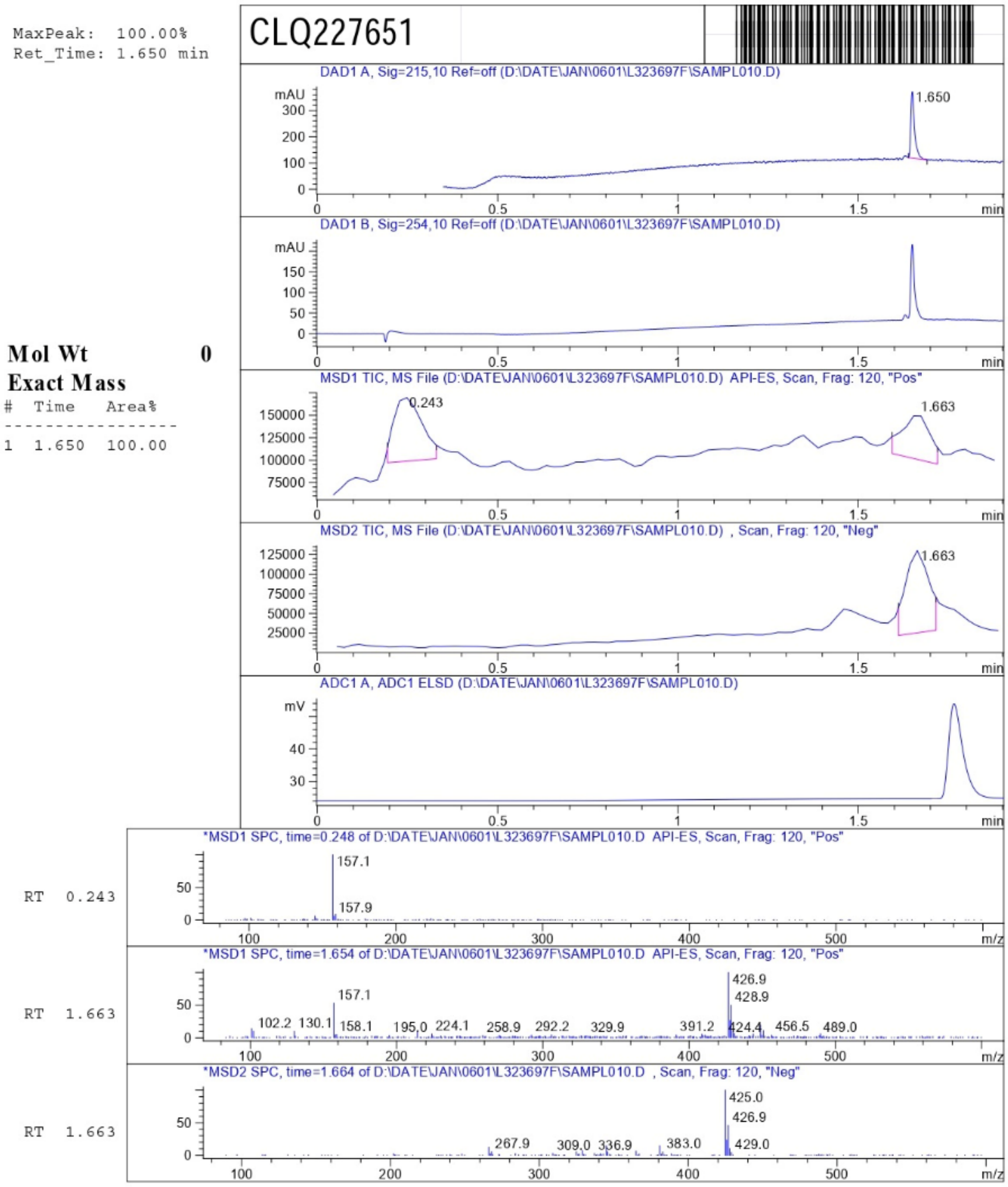

Figure S3. LC-MS spectrum of Les-3331

Crystal structure determination (X-ray) of Les-3331

In the salt crystal lattice of Les-3331 (Table S1) solute and solvent molecules related by translation along the a-axis are linked by hydrogen bonds O10—H10···O28, C15—H15···O9^{i}, C29—H29···O9 and C32—H32A···O9^{iii} into tapes (Figure S5, Table S2). The anti-parallel tapes, related by the center of symmetry, are then connected by hydrogen bonds C21—H21···O26^{ii} into columns (Figure S5, Table S2).

The carboxyl group present in the molecule, which is a fragment of the isobutylcarboxylic residue, forms a dihedral angle of 69.92(4)° with the mean plane of the 2-thioxo-1,3-thiazolidin-4-one system. The spatial orientation of this functional group is also determined by torsion angles C2–N3–C7–C8 and N3–C7–C8–O9 of 117.20(14) and –27.28(17)°, respectively. According to the values of the latter, bonds C2–N3 and C7–C8 take an anticlinal (+*ac*) conformation to each other, whereas bonds N3–C7 and C8–O9 an intermediate conformation between synperiplanar and synclinal (*sp*/–*sc*) ones. Spatial orientation of the remaining isobutyl fragment of the isobutylcarboxylic moiety is determined by torsion angles C2–N3–C7–C11, N3–C7–C11–C12 and N3–C7–C11–C13 of –112.25(15), 31.69(19) and 153.72(13)°, respectively. The values found to indicate that the C2–N3 and C7–C11 bonds adopt the anticlinal conformation (–*ac*), the N3–C7 and C11–C12 bonds an intermediate conformation between the synperiplanar and syncline (*sp*/+*sc*), and the N3–C7 and C11–C13 bonds an intermediate conformation between anticlinal and antipleriplanar (+*ac*/*ap*).The C5–C15 and C16–C18 bond lengths of 1.345(2) and 1.352(2) Å found in the 3-(4-nitrophenyl)-2-chloroprop-2-en-1-ylidene residue confirmed the presence of double bonds between these carbons. The mean planes of the 1,3-thiazolidine and phenyl rings present in the molecule form a dihedral angle of 16.91(4)°. The arrangement of atoms C15, C16, Cl17 and C18 belonging to the 3-(4-nitrophenyl)-2-chloroprop-2-en-1-ylidene moiety is flat (r.m.s.d. = 0.0008 Å) and lies almost in the plane of the 2-thioxo-1,3-thiazolidin-4-one system. The dihedral angle is only 1.76(6)°. Moreover, the mentioned arrangement of atoms C15, C16, Cl17 and C18 is positioned with respect to the phenyl ring at an angle of 15.44(6)°. The spatial arrangement of the 3-(4-nitrophenyl)-2-chloroprop-2-en-1-ylidene residue in the molecule is additionally determined by the torsion angles S1–C5–C15–C16, C5–C15–C16–C18 and Cl17–C16–C18–C19 of -1.8 (2), 179.34 (13) and -3.2(2)°, respectively, indicating the Z configuration of the bond pairs S1–C5/C15–C16 and Cl17–C16/C18–C19 and the *s-trans* conformation of the double bonds pair C5–C15/C16–C18. In the crystal, the conformation of the molecule is stabilized by the intra- and intermolecular hydrogen bonding (Figure S4 and Figure S5A, Table S2). The bond lengths O10—H10···O28, C29—H29···O9 and C32—H32A···O9^{iii} stabilize the spatial arrangement of the isobutylcarboxylic moiety whereas the hydrogen bonds C15—H15···O9^{i}, C21—H21···O26^{ii} and C24—H24···Cl17 stabilize the spatial arrangement of the 3-(4-nitrophenyl)-2-chloroprop-2-en-1-ylidene residue.

In the crystal lattice, solute molecules related by translation along the *a*-axis are linked by hydrogen bonds C15—H15···O9^{i} into tapes (Figure S4 and Figure S5A, Table S2). Solvent molecules do not participate in the formation of the tape but only attach to it through hydrogen bonds O10—H10···O28, C29—H29···O9 and C32—H32A·· O9^{iii}. The anti-parallel tapes, related by the center of symmetry, are connected by hydrogen bonds C21—H21·· O26^{ii} into columns (Figure S5B, Table S2).

Table S1. Crystal data, data collection and structure refinement for Les-3331

**Formula**	$C_{17}H_{15}N_2O_5S_2$, C_3H_7NO
**Formula weight**	499.97
**Temperature/K**	130.0(1)
**Wavelength/Å**	1.54184
**Crystal system**	triclinic
**Space group**	$P\bar{1}$
**Unit cell parameters (Å, º)**	a = 6.3403(2)
	b = 10.3925(4)
	c = 18.2404(7)
	α = 81.365(3)
	β = 84.987(3)
	γ = 76.138(3)
**Volume (Å³)**	1152.00(7)
Z **(*Z′*)**	2 (1)
D_c**/g cm**$^{-3}$	1.441
μ**/mm**$^{-1}$	3.530
F**(000)**	520
**Crystal dimensions [mm]**	0.42*0.13*0.04
**Color/Shape**	Yellow/lath
**Measurement method**	ω scans
θ **range for data collection (º)**	2.45–76.28
**Max/min. indices *h*, *k*, *l***	h: −7 → 7
	k: −12 → 12
	l: −22 → 21
**Collected reflections**	8975
**Independent reflections**	4666
R_{int}	0.0204
**Observed reflections [$I \geq 2\sigma(I)$]**	4352
**Completeness to θ_{max} = 76.28º /%**	97.2
**Completeness to θ_{full} = 67.68º /%**	99.9
**Restraints/Parameters**	0/297
**Abs. correction method**	Multi-scan
T_{min}, T_{max}	0.68721, 1.00000
**Goodness-of-fit on F^2**	1.060
**Final *R* indices [$I \geq 2\sigma(I)$]**	R1 = 0.0309, wR2 = 0.0823
***R* indices (all data)**	R1 = 0.0331, wR2 = 0.0847
**Largest diff. peak and hole /eÅ³**	0.336 and -0.284

Table S2. Hydrogen bonds in the crystal structure of Les-3331

D—H···*A*	*D*—H (Å)	H···*A* (Å)	*D*···*A* (Å)	*D*—H···*A* (°)
**O10—H10···O28**	0.90(3)	1.67(3)	2.5620(17)	169(3)
**C13—H13C···O10**	0.98	2.44	3.062(2)	121
**C15—H15···O9^{i}**	0.95	2.37	3.2218(17)	149
**C21—H21···O26^{ii}**	0.95	2.47	3.2175(18)	135
**C24—H24···Cl17**	0.95	2.53	3.2045(14)	128
**C29—H29···O9**	0.95	2.56	3.2280(18)	127
**C32—H32A···O9^{iii}**	0.98	2.58	3.527(2)	16159

Symmetry codes: (i) −1+x, y, z; (ii) −1−x, 2−y, 1−z; (iii) 1+x, y, z

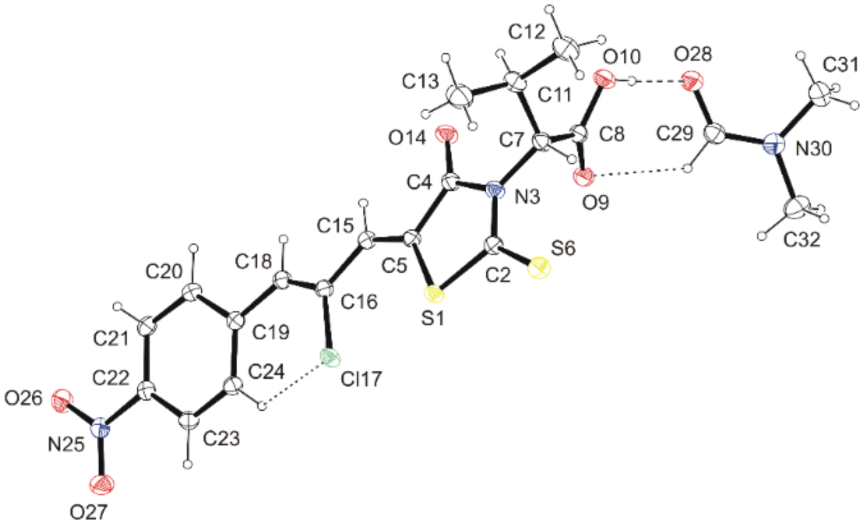

Figure S4. ORTEP view of Les-3331·DMF, showing the atomic labelling scheme. Non-H atoms are drawn as 30% probability displacement ellipsoids and H atoms are drawn as spheres of arbitrary size.

A)

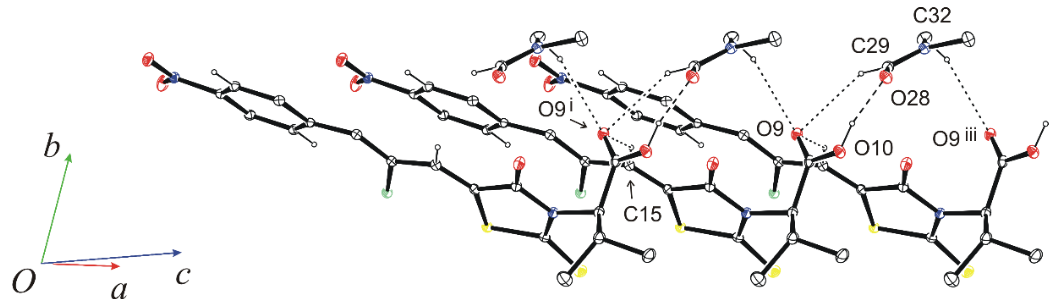

B)

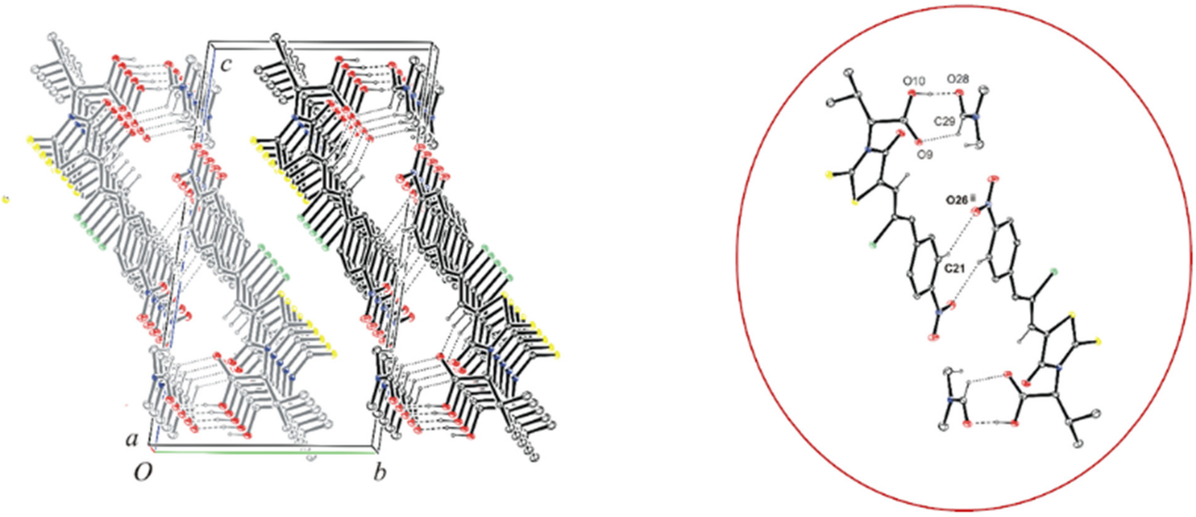

Figure S5. Hydrogen bonds linking molecules (**A**) into tapes growing along the a-axis, (**B**) linking inversion tapes into columns.

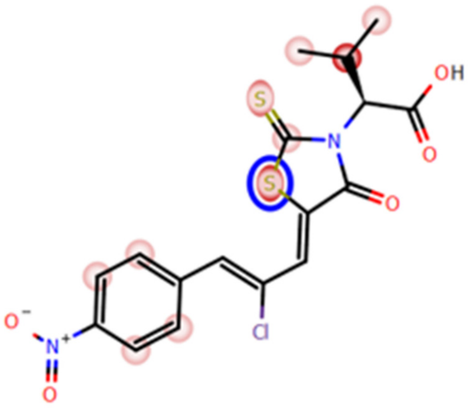

Figure S6. The MetaSite 8.0.1. *in silico* prediction of the most probable sites of Les-3331 metabolism

A)

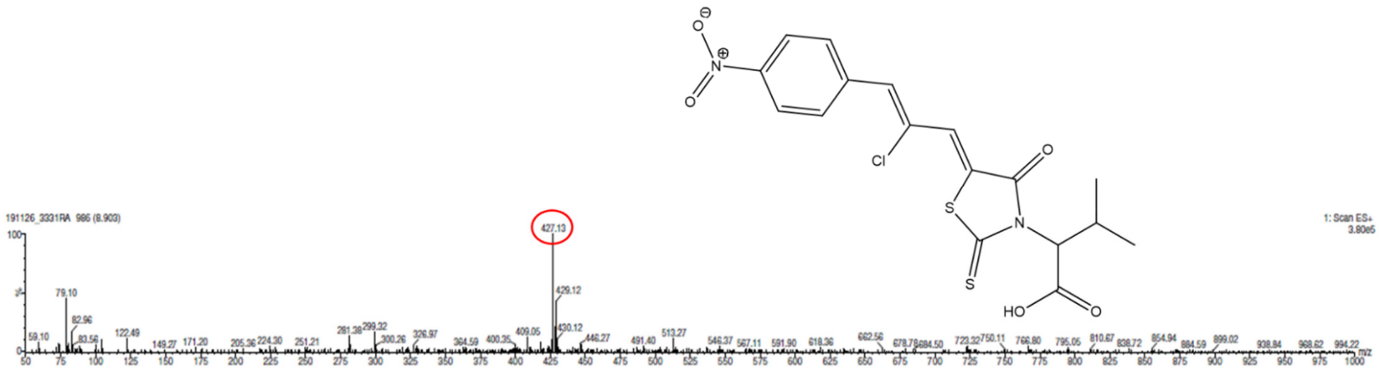

B)

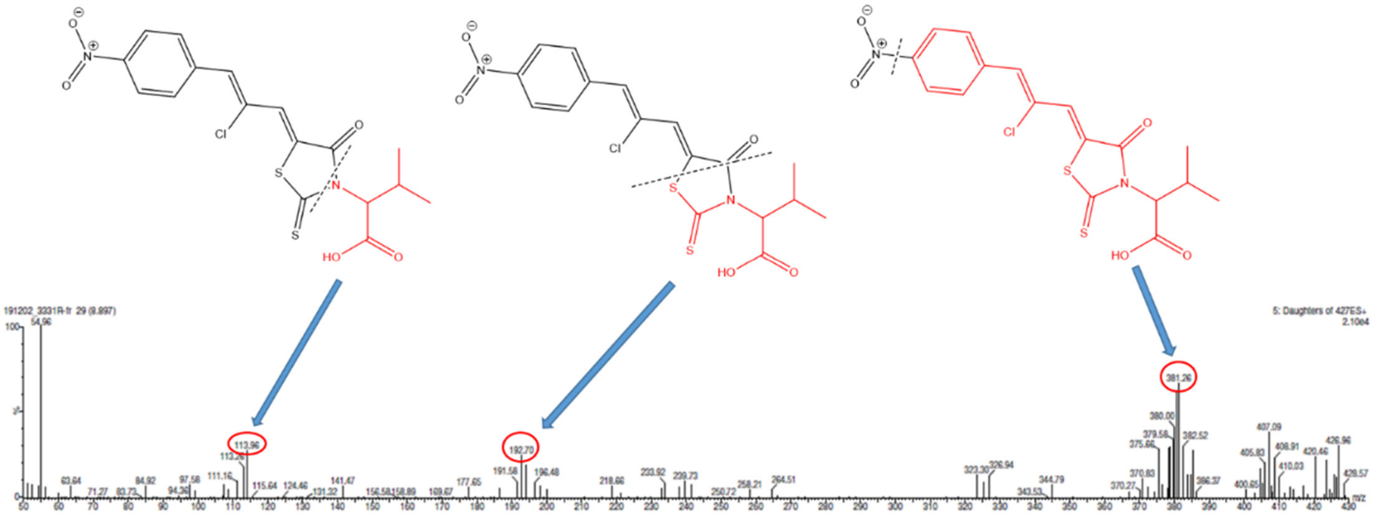

Figure S7. The MS spectrum (**A**) and MS ion fragment analyses (**B**) of compound Les-3331. The produced fragments of Les-3331 were marked in red.

A)

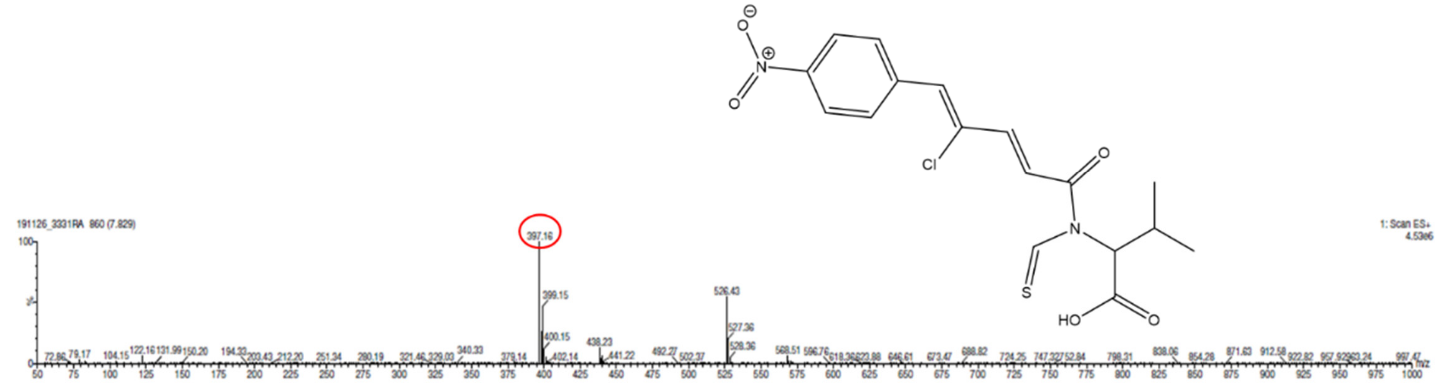

B)

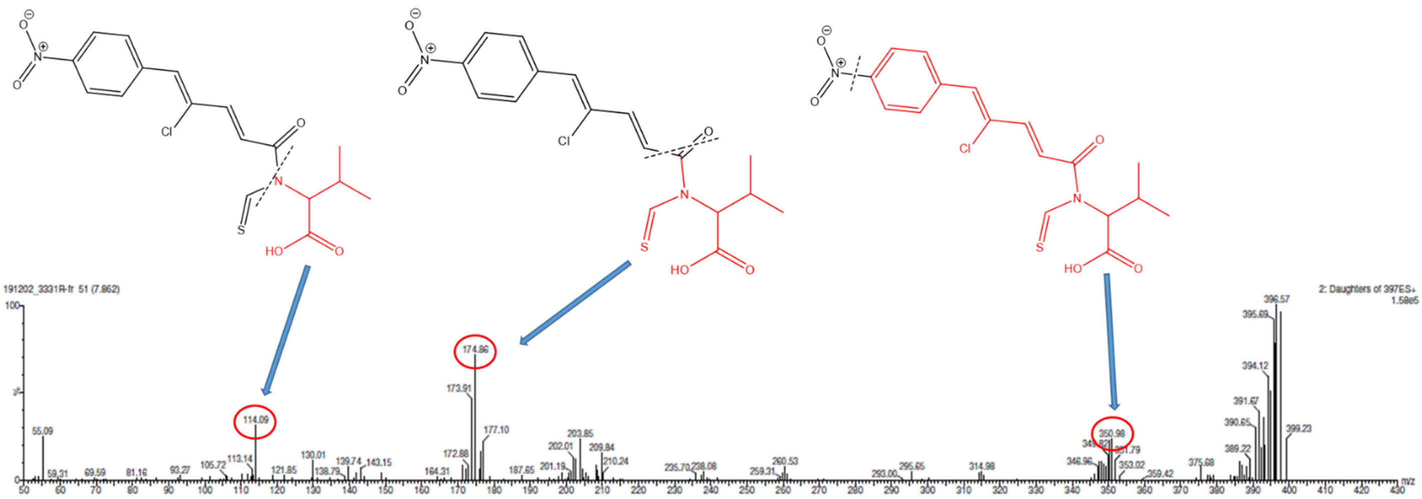

Figure S8. The MS spectrum and the most probable structure of Les-3331 main metabolite M1 (**A**). MS ion fragment analyses of compound Les-333 main metabolite M1 (**B**). The produced fragments were marked in red.

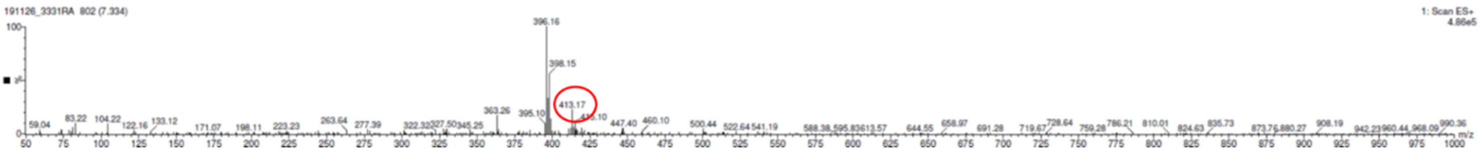

Figure S9. The MS spectrum of Les-3331 metabolite M2.

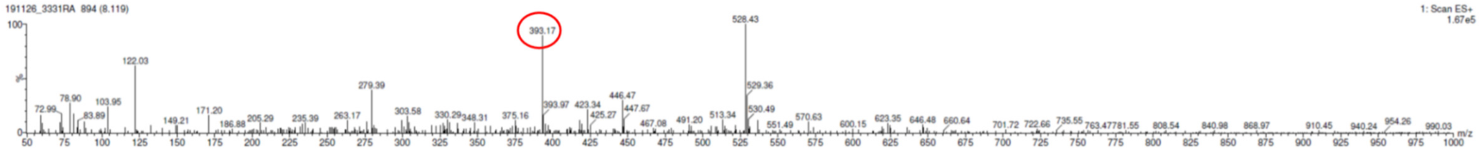

Figure S10. The MS spectrum of Les-3331 metabolite M3.

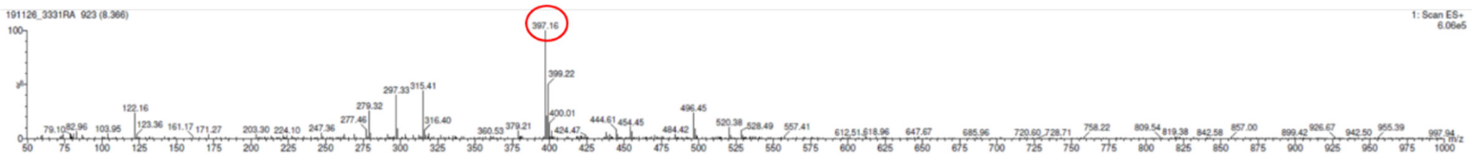

Figure S11. The MS spectrum of Les-3331 metabolite M4.

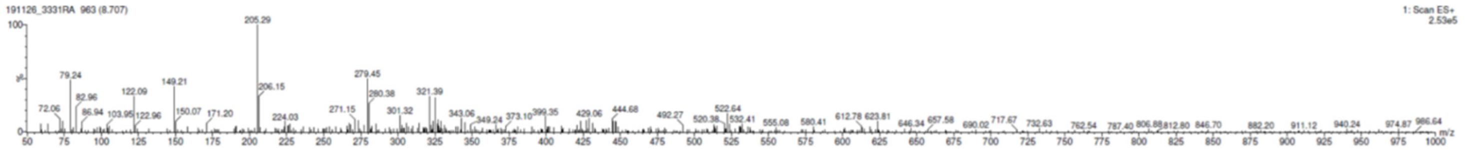

Figure S12. The MS spectrum of Les-3331 metabolite M5. Due to illegible data, the molecular weight of M5 was not estimated.

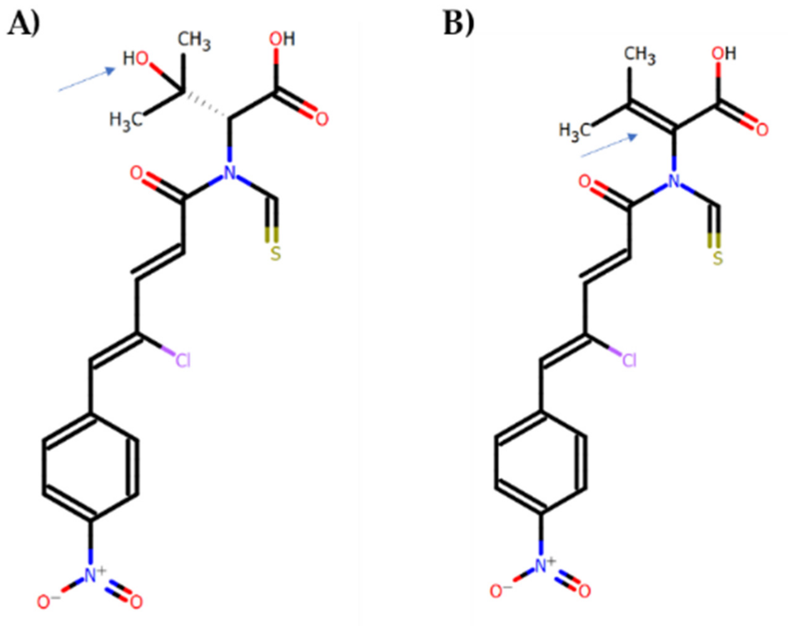

Figure S13. The MetaSite 8.0.1. *in silico* prediction of the most probable sites of the main metabolite M1 hydroxylation (A, metabolite M2) and dehydrogenation (B, metabolite M3).

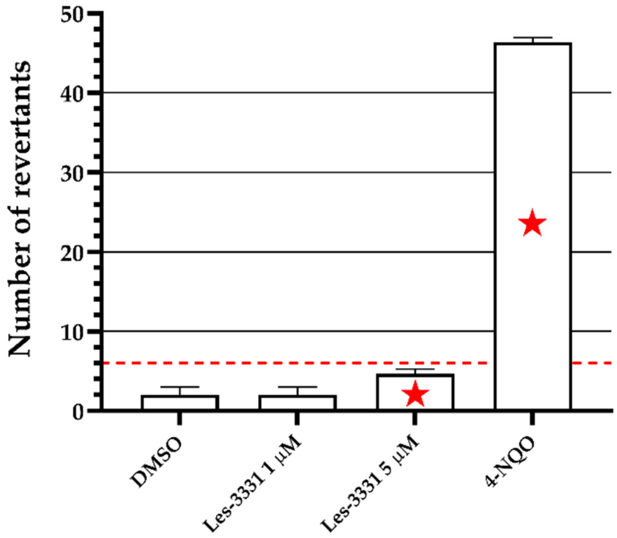

★Binomial B ≥ 0.99

Figure S14. The number of histidine prototrophic revertants of *Salmonella Typhimurium* strain TA100 exposed to the reference mutagen nonyl-4-hydroxyquinoline-N-oxide (4-NQO, 26.3 µM) and Les-3331 at 1 µM and 5 µM concentrations.

★ the Binomial B-value ≥ 0.99 and 2 ≥ fold increase of medium control baseline (dashed line) indicates the mutagenic effect
